# What to Do about Plastics? Lessons from a Study of United Kingdom Plastics Flows

**DOI:** 10.1021/acs.est.3c00263

**Published:** 2023-03-06

**Authors:** Michał P. Drewniok, Yunhu Gao, Jonathan M. Cullen, André Cabrera Serrenho

**Affiliations:** † School of Civil Engineering, Faculty of Engineering and Physical Sciences, University of Leeds, Woodhouse Ln., Woodhouse, LS2 9DY Leeds, United Kingdom; ‡ Department of Engineering, University of Cambridge, CB2 1PZ Cambridge, United Kingdom

**Keywords:** Plastics, Recycling, Demand, Stocks, Greenhouse gas emissions, United Kingdom, Trade

## Abstract

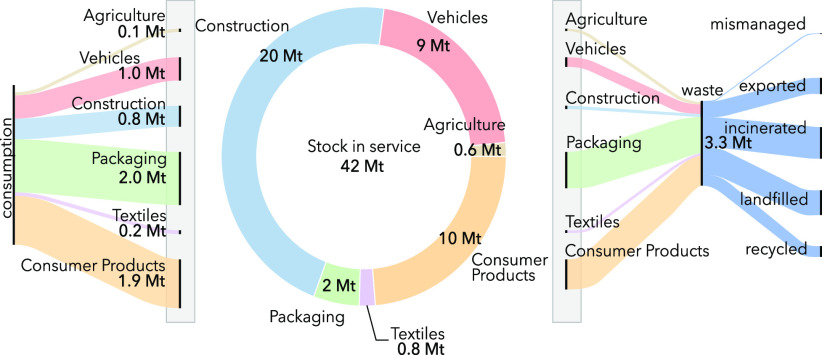

Plastics are one of the most widely used materials on the planet, owing to their usefulness, durability, and relatively low cost. Yet, making, using, and disposing of plastics create important environmental impacts, most notably greenhouse gas emissions and waste pollution. Reducing these impacts while still enjoying the benefits of plastic use requires an integrated assessment of all of the life cycles of plastics. This has rarely been attempted due to the wide variety of polymers and the lack of knowledge on the final uses and applications of plastics. Using trade statistics for 464 product codes, we have mapped the flows of the 11 most widely used polymers from production into six end-use applications for the United Kingdom (UK) in 2017. With a dynamic material flow analysis, we have anticipated demand and waste generation until 2050. We found that the demand for plastics seems to have saturated in the UK, with an annual demand of 6 Mt, responsible for approximately 26 Mt CO_2_*e*/a. Owing to a limited recycling capacity in the UK, only 12% of UK plastic waste is recycled domestically, leading to 21% of the waste being exported, labeled as recycling, but mostly to countries with poor practices of waste management. Increasing recycling capacity in the UK could both reduce GHG emissions and prevent waste pollution. This intervention should be complemented with improved practices in the production of primary plastics, which currently accounts for 80% of UK plastic emissions.

## Abstract 2

Mapping plastic flows in the United Kingdom shows that reduced United Kingdom recycling capacity is critical in limiting future reduction in United Kingdom plastic emissions and marine pollution.

## Introduction

The success of plastics is measured by their pervasiveness in society. The total global production of plastics, from the mid-20th century to today, equates to 9.2 billion tonnes.^1^ From this total, some 6.9 billion tonnes have already been discarded as waste, with 11% of this waste incinerated and 8% recycled.^1,2^ This immense amount of plastic material is made up of many different polymers, with a wide range of mechanical properties and colors, and used in a myriad of product applications at low cost.

However, the ubiquity of plastics has a significant impact on the environment, in the form of plastic waste in waterways and oceans and the release of greenhouse gas (GHG) emissions. In the 1960s, interactions between marine organisms and persistent litter were first observed and documented.^3^ By the early 1970s, plastic pellets were reported in the North Atlantic^4^ and eastern Pacific^5^ Ocean. More recently, Sir David Attenborough’s BBC Blue Planet II documentary^6^ shed fresh light on this problem, driving a change in the public perception of plastics.

GHG emissions are another important environmental impact of plastics. The modern petrochemical sector is responsible for 17% of global industrial GHG emissions,^7^ which is primarily released during the production of plastics and fertilizers. Emissions arise from chemical reactions and high temperature heat generation during the production of plastics, from energy conversion in the energy sector and from end-of-life waste management after the disposal of products made of plastics. Projections of a growing demand for plastics are likely to result in substantial additional GHG emissions, which is not compatible with existing climate targets.

In response to an increased public awareness of these problems, the European Union (EU) and United Kingdom (UK) policy-makers have recently announced bans on single-use plastics, such as plastic straws, stirrers, and plastic bags from 2021.^8,9^ In the UK, many stakeholders from the plastic value chains have signed the “UK Plastics Pact”,^10^ with commitments to achieve four targets by 2025: 100% reusable, recyclable, or compostable plastic packaging; 70% of plastic packaging effectively recycled or composted; eliminate single use packaging; 30% average recycled content across all plastic packaging.

Addressing the environmental impacts of plastics, while at the same time preserving the benefits of plastic products to users, requires a deeper understanding of the complex supply chain for polymers, products, and applications. However, as previously recognized by Levi and Cullen,^11^ detailed data on petrochemicals are scarce. Similarly, data on plastics production, use in manufactured products, and disposal are challenging to find, and for this reason, mapping the flows of plastics has rarely been attempted. One early attempt to map plastic flows was conducted by Joosten et al.,^12^ who mapped the flows of plastics in The Netherlands for 1990, using monetary transactions along the supply chain as a proxy to estimate physical material flows. More recently, Van Eygen et al.^13^ have performed a material flow analysis (MFA) for plastics in Austria for 2010, with a detailed allocation of plastics used across 11 end-use product categories. Both assessments were focused on the production, uses, and disposal of plastics as an aggregated material, but with only limited disaggregation by polymer.

Various attempts have been conducted to map the flows of specific groups of plastic polymers. Examples of this are the probabilistic MFA developed by Kawecki et al.,^14^ which has examined the flows of seven plastic polymers in Europe, and Di et al.,^15^ who have conducted a detailed material flow analysis for plastics in the USA for 2015, with flow disaggregation by polymer and including end-use product allocations and end-of-life waste flows. Other attempts have also accounted for the stocks of plastics in service, such as the MFA developed by Ciacci et al.,^16^ who have assessed the flows of PVC in Europe and estimated their stocks in service since 1960.

A handful of studies examine plastic production and use in the United Kingdom (UK), but are limited in scope to specific product segments or conducted at a highly aggregated level. For example, WRAP^17^ has estimated the flows of plastic packaging in the UK by conciliating several data sources. They estimate that in 2017 2.36 Mt of plastic packaging was placed on the UK market, of which 1.53 Mt was in the consumer sector. PlasticsEurope^18^ publishes annual aggregated statistics on plastic production, applications, and waste arisings in Europe and for some years for the UK. For 2016, PlasticsEurope^18^ estimates that 3.8 Mt of plastic postconsumer waste was collected through official schemes, 32% of which was sent to recycling, 38% incinerated, and the remaining 30% landfilled. Unfortunately, the narrow coverage and lack of depth for these studies limits their use in understanding the scale of plastic flows across the entire UK supply chain.

Extensive literature exists on the quantification of the environmental impacts of plastic production and disposal, for specific products or segments of the supply chain, in the form of life-cycle assessment (LCA) studies. For example, Chen et al.^19^ have provided a comprehensive LCA comparing the impacts of alternative end-of-life treatment options for plastics in China, including mechanical recycling, incineration and landfill. Other assessment have provided similar assessments for packaging waste in Portugal,^20^ or comparing recycling options in Italy.^21^ Some LCA studies have also explored alternative production routes of some of the resins used to produce plastic polymers, such as Ghanta et al.,^22^ who compared various routes for ethylene production. Several studies have assessed other impacts from plastic production and waste, including Azoulay et al.,^23^ who examined the impact of plastics on human health, and Jambeck et al.,^24^ who studied the impacts of the mismanagement of plastic waste.

Plastic production and disposal results in significant impacts from the release of GHG emissions. Zheng & Suh^25^ have quantified global GHG emissions from plastics across all stages of their life cycle. They have estimated that global emissions caused by plastics could increase from 1.7 Gt CO_2_*e* in 2015 to 6.5 Gt CO_2_*e* by 2050 if the sector continues the current trajectory of plastic demand and fails to change the way plastics are made. More recently, Nicholson et al.^26^ have quantified the energy and emissions associated with the plastics annually demanded by the USA. They have estimated that 104 Mt CO_2_*e* are produced annually to supply the current demand of plastics in the USA, and have highlighted the importance of reducing feedstock energy contributions for plastics manufacturing by shifting toward biobased and waste-based feedstock.

Many studies which assess the impacts of plastics, rarely take stock of plastic products currently in use, and how these stocks might evolve in the future. Such prospective assessments, which anticipate the future, are rare in literature, but critically important to identify the most meaningful opportunities for mitigating emissions. One exception is the ground-breaking study by Geyer et al.,^2^ which models the changes in global plastic stocks out to 2050. Using this dynamic model the authors estimate the future global demand for plastic products and the plastic waste arising from discarded products. The model anticipates an enormous quantity of plastic waste (12 Gt) being discarded to landfills or the natural environment by 2050. Similar analyses by Eriksen et al.^27^ and by Liu et al.^28^ have conducted a dynamic material flow analysis for PET, PE and PP in Europe, and for PVC in China, respectively.

Several studies have attempted to understand the flow of plastics along the supply chain, to quantify the environmental impacts of plastic production, use and disposal. However, we find that many studies are forced to trade-off the breadth of study scope with the depth of analysis, because of data limitations. On the one hand, assessments with a wide scope, taking in the whole life-cycle of plastics production, use and disposal, will often lack granular detail about specific polymers, products and applications. Four recent studies of plastic system in China,^29^ in Switzerland and Europe,^30^ and in the United States of America^15,31^ are the few exceptions. These provide historical detailed assessments of plastics flows, applications and disposal for a wide range of polymers. However, these studies do not explore the future, and the few prospective assessments conducted to date often aggregate flows into only a few polymers and applications. This lack of resolution makes the identification of specific interventions, particularly for product reuse and polymer recycling, challenging. On the other hand, assessments which focus on specific polymers or products (i.e., LCA analyses) or stages of the life-cycle (i.e., production or disposal) lack the breadth of analysis to understand the behavior of the whole plastics system and thus cannot weigh the true impact of intervention opportunities.

In this paper, we address this knowledge gap by developing a dynamic MFA that relates 464 product codes and disaggregates them by 11 polymers and 6 end-use product categories along all stages of the life cycle of plastics. This MFA is the first detailed map of UK plastic flows, which includes polymers, products, applications and trade flows. We use this model to test the impact of various interventions along the supply chain to reduce GHG emissions associated with the production and disposal of UK plastics. This allows us to identify priorities for intervention to reduce plastic waste production and to increase the value of plastic waste streams.

## Method: Modeling Current and Future Demand of Plastics and Waste Generation

A dynamic material flow analysis is required to test the impact of future interventions along the plastics supply chain in terms of GHG emissions and plastic waste generation. This analysis starts with an assessment of the flows of UK plastics from production to end-use products and waste generation, including imports and exports, since trade records exist and until 2017. This information is then used to model the dynamic of plastics flows, its accumulation into products in service and the later discard as waste. This model is then used to test the impact of future interventions along the supply chain of plastics in terms of waste production and global GHG emissions.

### Mapping Flows of Plastics in the UK

Data about plastics production, its end-use applications and waste generation is disperse, since there is no single publication or official statistics body providing systematic information about plastics in the supply chains. Detail and resolution about the individual polymers used at the various stages of the supply chain of plastics is even more challenging to find. Yet, this data is required to understand which polymers are used in plastic products and to anticipate the lifespan of products in service applications and when products will become available as waste.

The first task in mapping the flows of plastics is the collation of many disparate data sources, as presented in Table 1. Although some sources provide data on the production of UK plastics in primary form, no source is found to give estimates of plastics placed in service in the UK. This is a common problem of any material flow analysis, as statistics often report production flows but not where materials are used. Mapping the flows of plastics has an added difficulty, as it requires a prior estimate of the polymer content of all plastic products, the trade of polymers and plastic in and out of the UK, and their placement at the appropriate stages of the plastics supply chain.

**Table 1:** Source of Disparate Data Used for This Analysis

Flow	Source
Production	• production of plastics in primary form — PRODCOM^32^
	• plastics manufactured products — British Plastics Federation^33^
Export	• direct and indirect exports — PRODCOM^32^
Import	• direct and indirect imports — PRODCOM^32^
Waste	• waste generation by management route — PlasticsEurope^18^
	• packaging waste — WRAP^17^
	• recycling rates by polymer — WRAP^34^
	• PVC recycling rates — Recovinyl^35^

To collate this data for the UK, we require production and trade data. Although there are input-output (IO) tables for the UK, the derivation of physical flows from economic IO tables is subject to considerable uncertainty. Fortunately, PRODCOM trade data^32^ provides data in physical units for production and trade of a wide range of products, thus avoiding the additional uncertainty of converting economic data into physical data. We have thus identified 464 product categories from the PRODCOM^32^ as having some type of plastic in their composition. For each product category, we estimate the polymer composition and the plastic mass share, to build a picture of the aggregate mass of production, imports and exports for each product category and polymer type. Each product category is classified as belonging to one of the following stages of the supply chain: plastics in primary form, intermediates, end-use manufactured products, and waste. Production and trade flows are provided by PRODCOM^32^ and polymer composition data is obtained from the European data reported by PlasticsEurope.^18^ A detailed breakdown of this estimate is provided in Section 1 of the Supporting Information.

For waste generation, much fewer data is available. The main sources are PlasticsEurope,^18^ WRAP,^17,34,36^ and Eunomia.^37^ Further detail on plastic waste exports deemed to be recycled in other countries is obtained from PRODCOM^32^ and estimates on marine debris pollution and waste mismanagement is estimated from Jambeck et al.^24^ Full detailed data on waste generation is compiled in Section 2 of the Supporting Information.

### Understanding the Dynamic of Plastic Flows in the UK

Dynamic material flow analyses have been used successfully in several other studies to model the dynamics of material stocks in cars^38−40^ and buildings,^41,42^ but also in plastics.^2,27,28^ In this paper we use a similar method, starting from the mapping of plastic flows in the UK described before, disaggregated by end-use product categories. Each year (*n*), new plastics placed in service (*B*_in,*n*_) are required to meet the total demand for each end-use product category (*S*_*n*_), considering the plastics removed from service each year as waste (*B*_out,*n*_):
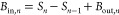
1

The amount of plastic waste generated each year (*B*_*out*,*n*_ in eq 1) is estimated by the failure rate of log-normal distribution for each cohort of end-use products with age *t* in each product category *p*. This is calculated using eq 2, where ϕ is the cumulative distribution function of the standard normal distribution. Our model uses the same life span distributions as Geyer et al.^2^ in their study of global plastics. Further details about the estimates of the distribution parameters *V* and *M* are provided in Section 4 of the Supporting Information.
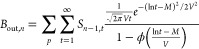
2

The input required to estimate future plastics demand and waste generation is the annual required stock of plastics (*S*_*n*_ in eq 1). This is obtained by multiplying stock per capita estimates with the UK Office for National Statistics’ projections for the future UK population.^43^ This is therefore a method of estimating future demand that is independent of any underlying economic model dependent on future GDP estimates. Although GDP growth may obviously influence the pace of increase in material accumulation for developing countries, most industrialized nations seem to have already the amount per capita of bulk materials they need. This has been shown by Pauliuk et al.^44^ for steel, and more recently by Wiedenhofer et al.^45^ for other materials. The method used to determine future stock per capita estimates required to compute *S*_*n*_ is described in detail in Section 5 of the Supporting Information.

### Quantifying the GHG Emissions Impact of Various Interventions

The dynamic model described in the previous section is used to explore the impact of possible future interventions to mitigate GHG emissions, along the whole life-cycle of plastics. This requires the use of estimates of emissions associated with production and disposal of various polymers and plastic products. Table 2 summarizes the emissions factors obtained from existing literature, which are used to build the emissions profiles in this analysis.

**Table 2:** Greenhouse Gas Emissions Considered in This Analysis for Various Polymers and Stages of Life Cycles. Landfill, recycling, and incineration emissions apply to all polymers.

Polymer	Production and conversion (kgCO_2_*e*/t)^25,46^		Landfill (kgCO_2_*e*/t)^25^	Recycling (kgCO_2_*e*/t)^25^	Incineration (kgCO_2_*e*/t)^25^
PET	2995		89	906	2351
PE-HD	2923				
PVC	2583				
PE-LD	2958				
PP	2806				
PS	2250				
PUR	4200				
PA	7000				
PMMA	4770				
PC	3400				
ABS	3100				
Others	3873				

The global emissions resulting from the production and disposal of plastics in the UK are estimated using the emission factors reported in Table 2. These factors are used in our dynamic model to quantify the future emissions of various interventions in the plastics supply chain. Table 3 presents a description of the three intervention options assessed. These seek to explore the potential to reduce GHG emissions of changes both upstream of plastics use (by reducing demand) and downstream (by increasing recycling). The effect of demand reduction is here exemplified only by a reduction in the demand for packaging, which is the single largest end-use fate of plastics in the UK.

**Table 3:** Tested Interventions in Supply Chain of Plastics in UK

Interventions	Description
(A) Business as usual	Continuation of trends verified in recent years: no change in per capita demand for all product categories (see Section 5 of Supporting Information for further evidence on this); no change in the split of waste by disposal destination and recycling capacity.
(B) Increasing UK recycling capacity	Linear increase in UK recycling capacity from current levels until 6 Mt in 2050 (which would be15 times greater than today), together with a linear change from the current share of end-of-life waste destinations to 90% recycling, 5% landfill, and 5% incineration in 2050.
(C) −50% demand for packaging per capita	Linear reduction in demand for packaging per capita from current 26 kg per capita to 13 kg per capita in 2050.

## Plastic Flows and Stocks in the UK

**Figure 1:**
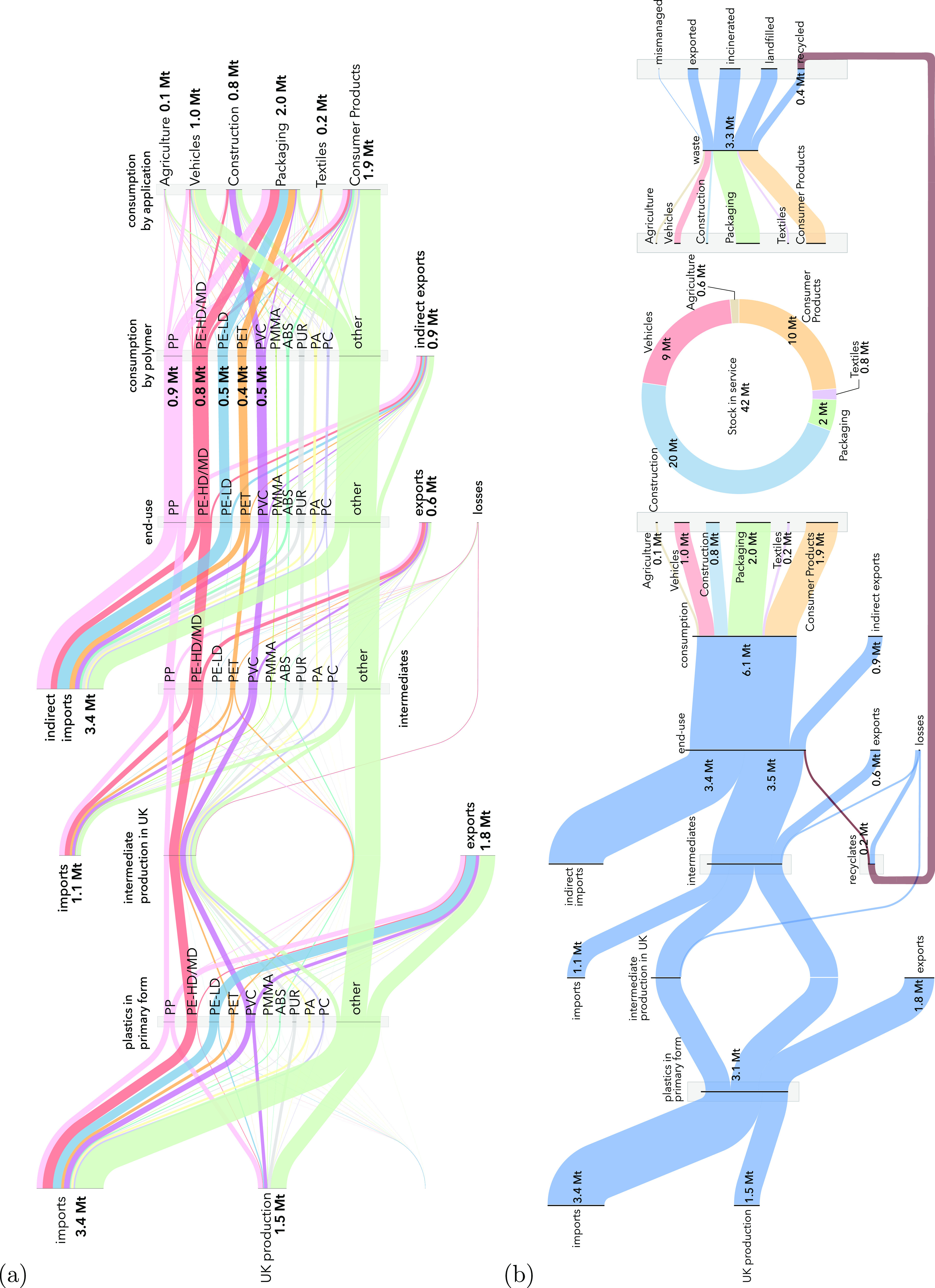
(a) Flows of plastics in the UK for 2017 from production of polymers in primary form to end-use products. (b) Summary of flows and stocks of plastics in the UK in 2017.

Figure 1(a) shows the modeled flows of the plastics from production of polymers in primary for to end-use products added to the service in the UK during 2017, using the method described in section ”Mapping ows of plastics in the UK”. The results show that in 2017 an estimated 6.3 Mt of plastics were consumed in the UK, most of which were imported as finished products and used as packaging (2.2 Mt) and consumer products (2.0 Mt). A wide range of polymers are used to supply UK demand, but PP, PE, PET and PVC together account for 58% of total demand.

**Figure 2:**
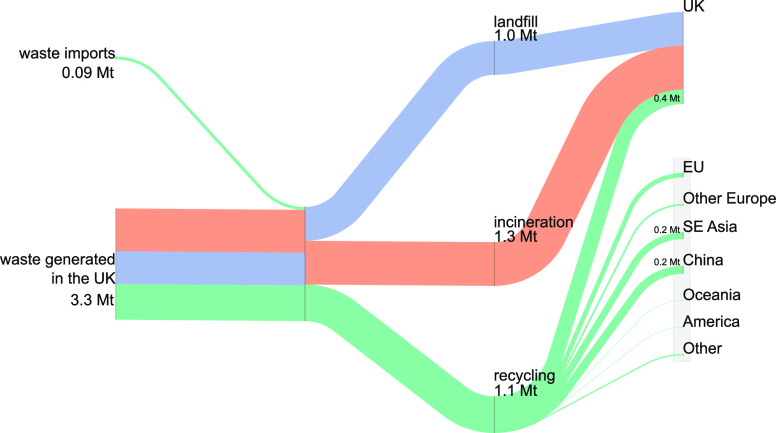
Fate of end-of-life UK plastic waste in 2017.

Plastic waste arisings are well reported by PlasticsEurope.^18^ However, understanding the final destination of UK waste requires a reconciliation of the data for waste arisings and waste trade state statistics.^47^ The results are shown in Figure 2 and reveal that of all plastic waste generated in the UK, approximately one-third is sent to landfill, one-third to incineration, and one-third for recycling. The limited recycling capacity in the UK (only approximately 0.4 Mt per year^34^) leads to 0.7 Mt of waste exports, labeled to be recycled in other countries. Yet, it is unclear whether exported plastic waste is effectively recycled at destination countries.

Our historical assessment of the dynamic of plastic flows in the UK enables the estimation of current stocks of plastics in service. The results are presented in Figure 1(b) and show that the stock of plastic products in service (42 Mt) is almost seven times the annual demand for plastics (6.3 Mt), owing to longer lifetimes of plastics used in construction and vehicles and consumer products. The limited amount of recycling capacity in the UK is also visible in Figure 1(b). Although recycling well-sorted waste streams of certain polymers is achieved with high yields of approximately 80%, lower recycling yields are observed for products containing mixed materials.^17^ High recycling yields are obtained for most plastic packaging waste and polymers, but average yield losses of 50% are reported^34^ for recycling nonpackaging waste. For this reason, we found that only 3% of the current demand for plastics in the UK is supplied by domestically recycled plastics. A detailed list of recycling yield estimates and references can be found in Section 3 of the Supporting Information.

## Impact of Interventions on Future Emissions and Waste Generation

**Figure 3:**
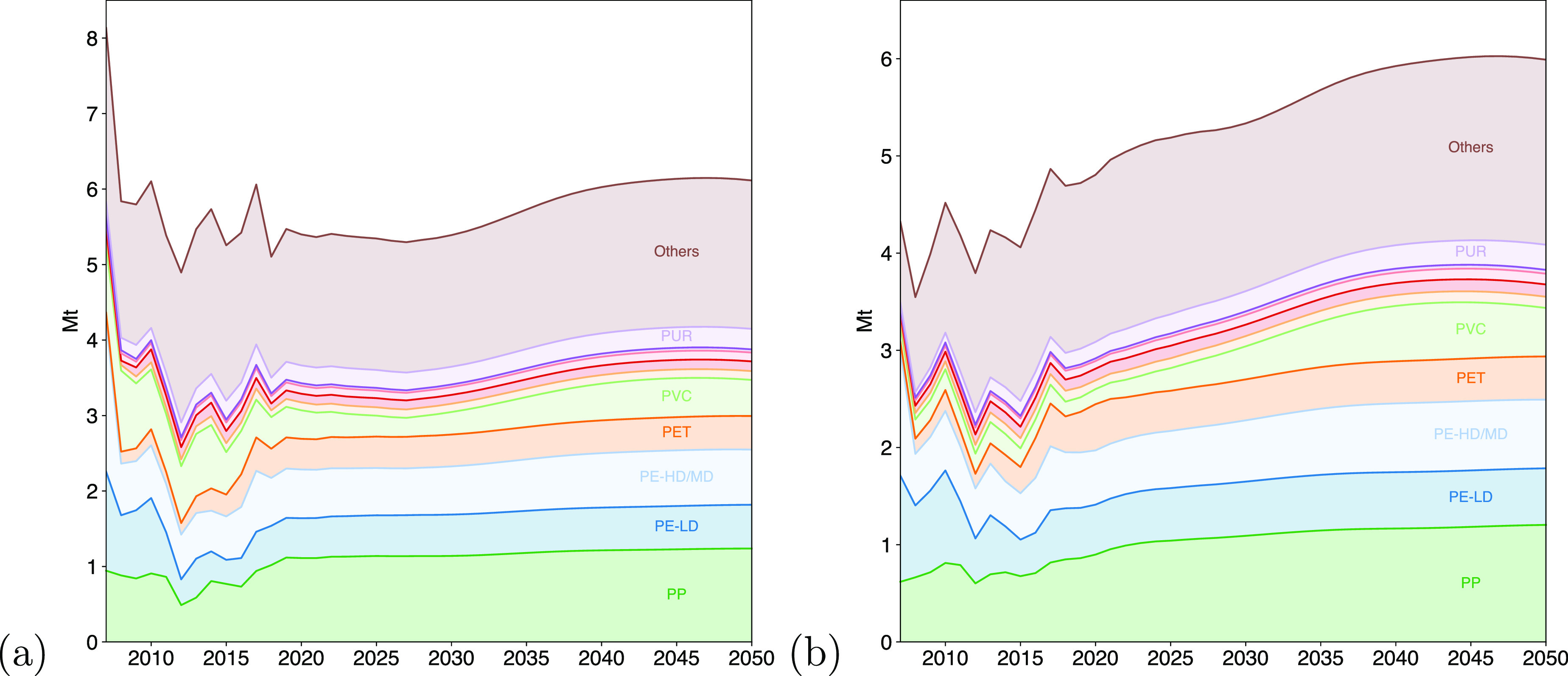
(a) Estimated demand for plastics in end-use products in the UK by polymer. (b) Estimated plastic waste generated in the UK by polymer.

The dynamic plastic flow model enables the estimation of the future demand for plastics in the UK. Figure 3(a) shows these results, where the demand for plastics is anticipated to remain stable through to 2050 at about 6 Mt of plastics per year. The proportion of demand in each product category and polymer is also expected to remain roughly constant, with the exception of construction, where an increase in demand is anticipated as a result of estimated new construction in the UK and the need to replace construction plastics installed over the last few decades.

The continued accumulation of plastics in service over the coming decades is expected to lead to an increase in the amount of plastic waste being generated in the UK. Figure 3(b) shows that if current patterns of plastic use continue, the UK will be required to treat approximately 50% more plastic waste in 2050 than today. Graphs similar to Figure 3 but with flows broken down by polymer are shown in Section 6 of Supporting Information.

**Figure 4:**
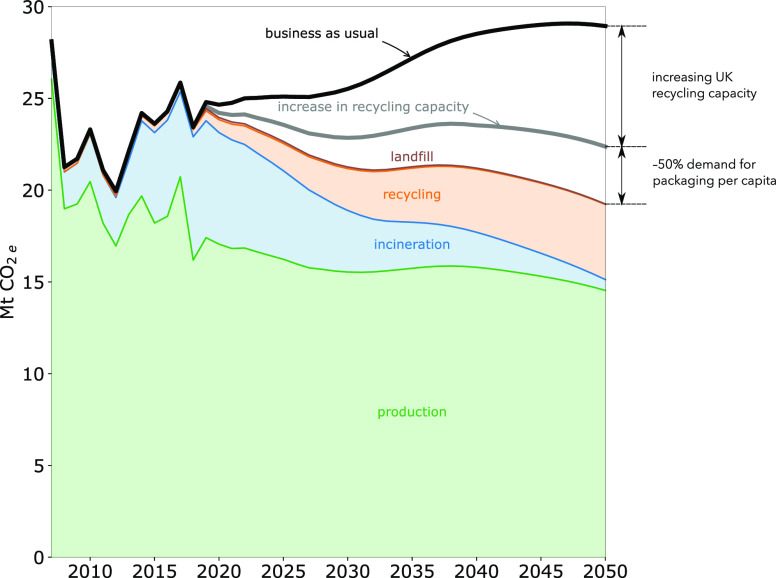
Estimated life cycle GHG emissions of UK plastics for each intervention listed in Table 3.

Figure 4 shows the resulting GHG emission reductions for each of the interventions listed in Table 3. Currently, two-thirds of the emissions associated with plastics used in the UK result from the production of polymers and manufacture of end-use plastic products, with the remaining third attributed almost entirely to the incineration of plastic waste in the UK. As a result, future reductions in plastic emissions will need to be based on a reduction of production and incineration emissions. For this reason, avoiding the production of virgin plastics, either by reducing demand or increasing plastic recycling, should be the basis of any mitigation interventions.

The expected increase in waste arisings shown in Figure 3(b) would result in a 16% increase in total UK plastics emissions, if there are no changes to the patterns of use and disposal (Figure 4). However, even with the joint implementation of interventions (B) and (C) from Table 3, only a modest decrease in total emissions would be achieved.

## Discussion

The results show that increasing recycling capacity in the UK (currently only 0.4 Mt per year^34^) could both avoid exported waste and avoid production of virgin plastics. This action alone would lead to a reduction of approximately 20% of UK plastics emissions in 2050, as suggested by the estimates shown in Figure 4.

Further reductions in the impacts associated with plastic production and disposal would require a reduction in the demand for plastics. Currently, packaging accounts for approximately 40% of the annual consumption of plastics in the UK and having this demand alone could lead to an important but modest reduction in GHG emissions, as shown in Figure 4. However, a reduction in the demand for packaging plastics would lead to a rapid reduction in in the environmental impacts of plastic waste management, owing to the short service lives of packaging products. According to WRAP,^17^ approximately 35% of plastic packaging used annually in the UK market is packaging used in business-to-business transactions. Reducing this type of packaging could be a meaningful opportunity to reduce total packaging demand without directly impacting on final consumers and their purchasing behavior.

However, Figure 4 shows that the combined potential for emissions savings of the above measures (interventions (B) and (C) from Table 3), although important, only account for approximately one-third of 2050 UK plastics emissions. This is because of high yield losses in plastic recycling, owing to polymer mixing in waste streams and the limitations of the mechanical recycling processes. As a result, a substantial increase in recycling capacity would not directly translate to an offset in the production of virgin polymers and their associated emissions.

Further emissions savings would have to be generated from a combination of other strategies, including improved product design to enable longer product lives, increased reusability, and easier separation from waste streams. Polymers produced from biomass feedstock instead of fossil fuels could be an effective alternative to reduce environmental impacts associated with the production of plastics. However, biobased plastics create currently important challenges to plastic waste management systems, as these are designed to separate and recycle conventional plastics,^48^ and the impacts of biobased plastics in the environment are not always known. Moreover, some biobased plastics can lead to more disposal GHG emissions than conventional plastics, as was exemplified by Papong et al.^49^ who concluded that landfilling PLA can produce more emissions than incinerating PET. Additionally, biomass feedstock production is likely to compete with food production for land use, which may prevent the deployment of these materials at scale, and the adoption of alternative materials needs to be able to compete with the current low costs of plastic production.

The projections presented in this study consider current practices in the chemical industry and energy sector. Decarbonization of the electricity supply and petrochemical processes may reduce production emissions of plastics but does not change the impacts of waste management and waste pollution. However, innovative recycling technologies, such as chemical and feedstock recycling, could enable higher grades of recycled plastics and lower yield losses, thus increasing the potential of recycling as an effective strategy to reduce waste and emissions. Chemical recycling is still not available at commercial scale, but if powered with zero-emission energy sources, it could lead to the displacement of virgin plastic production and substantial emissions savings.

The analysis devised in this study relies on quantitative data on mass flows from PRODCOM trade statistics^32^ and granular data on polymer use by product applications from European trade association statistical data.^18^ Despite the use of uncommon top-down data sources, our MFA estimates produced results in line with various existing plastics MFA for other countries. We estimated a current annual consumption of approximately 0.1 t per capita, which is of the same order of magnitude of other studies, such as 0.156 t per capita for Austria in 2010,^13^ 0.11 t per capita for the USA in 2015,^15^ and twice the value estimated by Jiang et al.^29^ for China in 2017. Similarly, our shares of plastic consumption by end-use application show the predominance of packaging, followed by vehicles, construction, and consumer products, which has also been observed globally by Geyer et al.^2^ and at the European level by PlasticsEurope.^18^ We have also validated our model by comparing the model outputs on plastic consumption and waste generation with other disparate estimates available in the literature for the UK, such as WRAP^17^ and Eunomia.^37^ The validation shows a good agreement with other existing estimates, with the details included in Section 7 of the Supporting Information.

The results provided in this study result from reconciling disparate data sources about plastics at various stages of the supply chain and with different levels of granularity and frequency of reporting, which do not have assigned uncertainties. For this reason, it is challenging to perform a formal uncertainty analysis to the estimates presented in this study. However, all data used in this study were extracted from official statistics (e.g., PRODCOM), plastics trade associations (e.g., PlasticsEurope), peer-reviewed academic literature, and estimates provided by knowledgeable specialists (e.g., Recovinyl). The validation of our results by comparison with other similar studies for other countries shows that the uncertainties associated with our estimates do not appear to compromise the order of magnitude and scale of the key destinations of plastic demand and sources of waste generation, and this is sufficient to support the claims made in this section. However, the lack of detailed data on polymer composition for all 464 PRODCOM products codes considered in this analysis, and particularly for the codes accounting for the smallest mass flows, is an important source of uncertainty, and therefore our estimates should not be considered as providing detailed polymer compositions for all product categories. A detailed discussion on our approach to coverage and resolution of product categories is provided in Section 1 of the Supporting Information.

There are no official data sets reporting regularly and with enough resolution about the production, use, and disposal of plastics. This has been one of the key challenges for undertaking material flow analyses and for understanding the uncertainty and variability of results. For this reason, we share the conclusions reported by Wang et al.^50^ on the need for research addressing the problems of inconsistent classification, missing data, conflicting data, and inexplicit data for plastics products and waste. We hope that the potential for novel insights capable of mitigating most of the environmental impacts of plastics and other materials could be a strong incentive to enhance data acquisition and reporting of resource flows.

## Supporting Information Available

Additional details of the dynamic model, including data on polymer composition, end-of-life estimates, and validation of results (PDF)

## References

[ref1] Letcher T. Plastic Waste and Recycling: Environmental Impact, Societal Issues, Prevention, and Solutions; Elsevier Science & Technology, 2020.

[ref2] Geyer R.; Jambeck J. R.; Law K. L. Production, use, and fate of all plastics ever made. Science Advances 2017, 3, e1700782 10.1126/sciadv.1700782. 28776036 PMC5517107

[ref3] Ryan P. G. Marine Anthropogenic Litter; Springer: Cham, 2015; pp 1–25.

[ref4] Carpenter E. J.; Smith K. Plastics on the Sargasso Sea surface. Science 1972, 175, 1240–1241. 10.1126/science.175.4027.1240. 5061243

[ref5] Wong C.; Green D. R.; Cretney W. J. Quantitative tar and plastic waste distributions in the Pacific Ocean. Nature 1974, 247, 30–32. 10.1038/247030a0.

[ref6] Blue Planet II documentary. BBC, 2017; https://www.bbc.co.uk/programmes/p04tjbtx (accessed 2023–02–20).

[ref7] World Energy Balances; International Energy Agency: Paris, France, 2019.

[ref8] The Environmental Protection (Plastic Straws, Cotton Buds and Stirrers) (England) Regulations 2020; UK Statutory Instrument 2020, No. 971; 2020.

[ref9] Directive (EU) 2019/904 of the European Parliament and of the Council of 5 June 2019 on the reduction of the impact of certain plastic products on the environment (Text with EEA relevance). Official Journal of the European Union, 2019.

[ref10] The UK Plastics Pact. WRAP. https://www.wrap.org.uk/content/the-uk-plastics-pact (accessed 2021-02–10).

[ref11] Levi P. G.; Cullen J. M. Mapping Global Flows of Chemicals: From Fossil Fuel Feedstocks to Chemical Products. Environ. Sci. Technol. 2018, 52, 1725–1734. 10.1021/acs.est.7b04573. 29363951

[ref12] Joosten L. A. J.; Hekkert M. P.; Worrell E. Assessment of the plastic flows in The Netherlands using STREAMS. Resources, Conservation and Recycling 2000, 30, 135–161. 10.1016/S0921-3449(00)00055-0.

[ref13] Van Eygen E.; Feketitsch J.; Laner D.; Rechberger H.; Fellner J. Comprehensive analysis and quantification of national plastic flows: The case of Austria. Resources, Conservation and Recycling 2017, 117, 183–194. 10.1016/j.resconrec.2016.10.017.

[ref14] Kawecki D.; Scheeder P. R. W.; Nowack B. Probabilistic Material Flow Analysis of Seven Commodity Plastics in Europe. Environ. Sci. Technol. 2018, 52, 9874–9888. 10.1021/acs.est.8b01513. 30004221

[ref15] Di J.; Reck B. K.; Miatto A.; Graedel T. E. United States plastics: Large flows, short lifetimes, and negligible recycling. Resources, Conservation and Recycling 2021, 167, 105440 10.1016/j.resconrec.2021.105440.

[ref16] Ciacci L.; Passarini F.; Vassura I. The European PVC cycle: In-use stock and flows. Resources, Conservation and Recycling 2017, 123, 108–116. 10.1016/j.resconrec.2016.08.008.

[ref17] PlasticFlow 2025, Plastic Packaging Flow Data Report; WRAP, 2018.

[ref18] Plastics – The Facts 2019, An Analysis of European Plastics Production, Demand and Waste Data; PlasticsEurope, 2019.

[ref19] Chen Y.; Cui Z.; Cui X.; Liu W.; Wang X.; Li X.; Li S. Life cycle assessment of end-of-life treatments of waste plastics in China. Resources, Conservation and Recycling 2019, 146, 348–357. 10.1016/j.resconrec.2019.03.011.

[ref20] Ferreira S.; Cabral M.; da Cruz N. F.; Simões P.; Marques R. C. Life cycle assessment of a packaging waste recycling system in Portugal. Waste Management 2014, 34, 1725–1735. 10.1016/j.wasman.2014.05.007. 24910140

[ref21] Perugini F.; Mastellone M. L.; Arena U. A life cycle assessment of mechanical and feedstock recycling options for management of plastic packaging wastes. Environmental Progress 2005, 24, 137–154. 10.1002/ep.10078.

[ref22] Ghanta M.; Fahey D.; Subramaniam B. Environmental impacts of ethylene production from diverse feedstocks and energy sources. Appl. Petrochem. Res. 2014, 4, 167–179. 10.1007/s13203-013-0029-7.

[ref23] Azoulay D.; Villa P.; Arellano Y.; Gordon M.; Moon D.; Miller K.; Thompson K. Plastic & Health: The Hidden Costs of a Plastic Planet; Center for International Environmental Law (CIEL), 2019.

[ref24] Jambeck J. R.; Geyer R.; Wilcox C.; Siegler T. R.; Perryman M.; Andrady A.; Narayan R.; Law K. L. Plastic waste inputs from land into the ocean. Science 2015, 347, 768 10.1126/science.1260352. 25678662

[ref25] Zheng J.; Suh S. Strategies to reduce the global carbon footprint of plastics. Nature Climate Change 2019, 9, 374–378. 10.1038/s41558-019-0459-z.

[ref26] Nicholson S. R.; Rorrer N. A.; Carpenter A. C.; Beckham G. T. Manufacturing energy and greenhouse gas emissions associated with plastics consumption. Joule 2021, 5, 673–686. 10.1016/j.joule.2020.12.027.

[ref27] Eriksen M. K.; Pivnenko K.; Faraca G.; Boldrin A.; Astrup T. F. Dynamic Material Flow Analysis of PET, PE, and PP Flows in Europe: Evaluation of the Potential for Circular Economy. Environ. Sci. Technol. 2020, 54, 16166–16175. 10.1021/acs.est.0c03435. 33225689

[ref28] Liu Y.; Zhou C.; Li F.; Liu H.; Yang J. Stocks and flows of polyvinyl chloride (PVC) in China: 1980–2050. Resources, Conservation and Recycling 2020, 154, 104584 10.1016/j.resconrec.2019.104584.

[ref29] Jiang X.; Wang T.; Jiang M.; Xu M.; Yu Y.; Guo B.; Chen D.; Hu S.; Jiang J.; Zhang Y.; Zhu B. Assessment of Plastic Stocks and Flows in China: 1978–2017. Resources, Conservation and Recycling 2020, 161, 104969 10.1016/j.resconrec.2020.104969.

[ref30] Kawecki D.; Wu Q.; Gonçalves J. S. V.; Nowack B. Polymer-specific dynamic probabilistic material flow analysis of seven polymers in Europe from 1950 to 2016. Resources, Conservation and Recycling 2021, 173, 105733 10.1016/j.resconrec.2021.105733.

[ref31] Heller M. C.; Mazor M. H.; Keoleian G. A. Plastics in the US: toward a material flow characterization of production, markets and end of life. Environmental Research Letters 2020, 15, 094034 10.1088/1748-9326/ab9e1e.

[ref32] Prodcom statistics. Eurostat. https://https://ec.europa.eu/eurostat/web/prodcom (accessed 2021–02–10).

[ref33] About The British Plastics Industry. BPF. https://www.bpf.co.uk/industry/Default.aspx (accessed 2021–02–10).

[ref34] Plastics Market Situation Report 2019; One Planet, 2019.

[ref35] Recovinyl. https://www.recovinyl.com (accessed 2021-02–10).

[ref36] UK Plastics Waste – A review of supplies for recycling, global market demand, future trends and associated risks.; WRAP, 2006.

[ref37] Plastic Packaging. Shedding Light on the UK Data; Eunomia, 2018.

[ref38] Cabrera Serrenho A.; Allwood J. M. Material Stock Demographics: Cars in Great Britain. Environ. Sci. Technol. 2016, 50, 3002–3009. 10.1021/acs.est.5b05012. 26871002

[ref39] Serrenho A. C.; Norman J. B.; Allwood J. M. The impact of reducing car weight on global emissions: the future fleet in Great Britain. Philosophical Transactions of the Royal Society A: Mathematical, Physical and Engineering Sciences 2017, 375, 20160364 10.1098/rsta.2016.0364. PMC5415645 28461428

[ref40] Craglia M.; Cullen J. Modelling transport emissions in an uncertain future: What actions make a difference?. Transportation Research Part D: Transport and Environment 2020, 89, 102614 10.1016/j.trd.2020.102614.

[ref41] Cabrera Serrenho A.; Drewniok M.ł.; Dunant C.; Allwood J. M. Testing the greenhouse gas emissions reduction potential of alternative strategies for the english housing stock. Resources, Conservation and Recycling 2019, 144, 267–275. 10.1016/j.resconrec.2019.02.001.

[ref42] Dunant C. F.; Shah T.; Drewniok M. P.; Craglia M.; Cullen J. M. A new method to estimate the lifetime of long-life product categories. Journal of Industrial Ecology 2021, 25, 321–332. 10.1111/jiec.13093.

[ref43] Office for National Statistics, 2017; https://www.ons.gov.uk/peoplepopulationandcommunity/populationandmigration/populationprojections (accessed 2021–02–10).

[ref44] Pauliuk S.; Milford R. L.; Müller D. B.; Allwood J. M. The steel scrap age. Environ. Sci. Technol. 2013, 47, 3448–54. 10.1021/es303149z. 23442209

[ref45] Wiedenhofer D.; Fishman T.; Plank B.; Miatto A.; Lauk C.; Haas W.; Haberl H.; Krausmann F. Prospects for a saturation of humanity’s resource use? An analysis of material stocks and flows in nine world regions from 1900 to 2035. Global Environmental Change 2021, 71, 102410 10.1016/j.gloenvcha.2021.102410.

[ref46] Europe Ecoprofiles, 2019. PlasticsEurope. https://www.plasticseurope.org/en/resources/eco-profiles (accessed 2021–02–10).

[ref47] UK Trade Info, 2019. HMRC. https://www.uktradeinfo.com/ (accessed 2021–02–10).

[ref48] Gerassimidou S.; Martin O. V.; Chapman S. P.; Hahladakis J. N.; Iacovidou E. Development of an integrated sustainability matrix to depict challenges and trade-offs of introducing bio-based plastics in the food packaging value chain. Journal of Cleaner Production 2021, 286, 125378 10.1016/j.jclepro.2020.125378.

[ref49] Papong S.; Malakul P.; Trungkavashirakun R.; Wenunun P.; Chom-in T.; Nithitanakul M.; Sarobol E. Comparative assessment of the environmental profile of PLA and PET drinking water bottles from a life cycle perspective. Journal of Cleaner Production 2014, 65, 539–550. 10.1016/j.jclepro.2013.09.030.

[ref50] Wang C.; Liu Y.; Chen W.-Q.; Zhu B.; Qu S.; Xu M. Critical review of global plastics stock and flow data. Journal of Industrial Ecology 2021, 25, 1300–1317. 10.1111/jiec.13125.

