# Supplementary material for: What to Do about Plastics? Lessons from a Study of United Kingdom Plastics Flows
Source: Environ Sci Technol. 2023 Mar 6;57(11):4513–21. doi: 10.1021/acs.est.3c00263 (PMC10035030; doi:10.1021/acs.est.3c00263)
Supplement: Supplementary file 1 — es3c00263_si_001.pdf [file es3c00263_si_001.pdf]

SUPPLEMENTARY INFORMATION FILE

WHAT TO DO ABOUT PLASTICS? LESSONS FROM A STUDY OF UNITED KINGDOM PLASTICS FLOWS

Michał Drewniok, Yunhu Gao, Jonathan M. Cullen, André Cabrera Serrenho

Department of Engineering, University of Cambridge, United Kingdom

Summary: 35 pages, 10 figures, 9 tables.

Data

Input, output data, and Python code files can be accessed online at: https://doi.org/10.17863/CAM.92501

Table of Contents

Data 1

1. Polymer composition for PRODCOM product categories 2

2. End-of-life waste estimates 26

3. Recycling yields 28

4. Log-Normal parameter estimation 30

5. Demand estimates 30

6. Estimates of plastics demand and waste generation 33

7. Validation 34

References 35

1. Polymer composition for PRODCOM product categories

There are 464 product categories in PRODCOM (Eurostat, 2018) for products that either contain or are made of any plastic. Each of these categories was systematically classified according to the following aspects:

1. Stage of supply chain — whether it is a plastic in primary form, an intermediate used by manufacturers, an end-use product, or a waste flow.
2. Mass fraction of plastics — this implied estimating the fraction of the mass of each product category with is made of plastics. Prior to this, if the quantity is not reported by PRODCOM in mass units, it was necessary to convert the reported units to mass units.
3. Polymer composition — this implied the estimation of the mass fraction of each polymer.
4. End-use application — for each product category that is not a plastic in primary form, we estimated the allocation of the mass share of each polymer to end-use product categories. This was informed by the allocation matrix of polymers to end-use applications published by PlasticsEurope (PlasticsEurope, 2019). The detailed classification by end-use application is shown in section in the file 'Polymer allocation.xlsx' in our data repository available at: https://doi.org/10.17863/CAM.92501.

Table S 1 details the classifications of all PRODCOM categories by stage of supply chain, mass fraction of plastics, and polymer composition. This table can be accessed in xlsx format online at https://doi.org/10.17863/CAM.92501

For many PRODCOM product codes we could not find a detailed polymer composition. For these codes we have defined an end-use application as defined in Table S 2. In these cases, we considered the average polymer composition for each end-use application as reported by PlasticsEurope (PlasticsEurope, 2019), and reproduced here in Table S 3. The allocations (by polymer and by end-us application) reproduced in this section, along with the Python code we used, are also available online at https://doi.org/10.17863/CAM.92501. These tables should therefore not be considered as detailed compositions for all product codes.

Table S 1. Classification of each code of PRODCOM product categories. 'Code' is the reference code for product categories in PRODCOM (Eurostat, 2018). 'Stage of supply chain' indicates the stage at which each product is considered; 'P' stands for plastics in primary form; 'C' for intermediates; 'E' for end-use products. 'Unit conversion coefficient' is the value which multiplied by the flow value in PRODCOM results in the mass in kg. Polymer acronyms: PE-LD= low density polyethylene, PE-HD/MD=high or medium density polyethylene, PP=polypropylene, PVC=polyvinyl chloride, PET=polyethylene terephthalate, PS=polystyrene, EPS=expanded polystyrene, ABS=acrylonitrile butadiene styrene, PA=polyamide, PC=polycarbonate, PMMA=poly(methyl methacrylate), PUR= polyurethane, O = other

				Mass fraction of each polymer												
Code	Stage of supply chain	Unit conversion coefficient	Plastics mass fraction	PE-LD	PE-HD/MD	PP	PVC	PET	PS	EPS	ABS	PA	PC	PMMA	PUR	O
10511133	E	1.000	0.029		1.00											
10511137	E	1.000	0.016		1.00											
10511142	E	1.000	0.029		1.00											
10511148	E	1.000	0.016		1.00											
10511210	E	1.000	0.029		1.00											

				Mass fraction of each polymer												
Code	Stage of supply chain	Unit conversion coefficient	Plastics mass fraction	PE-LD	PE-HD/MD	PP	PVC	PET	PS	EPS	ABS	PA	PC	PMMA	PUR	O
10511220	E	1.000	0.016		1.00											
10511230	E	1.000	0.029		1.00											
10511240	E	1.000	0.016		1.00											
10512230	E	1.000	0.009		1.00											
10512260	E	1.000	0.003		1.00											
10515245	E	1.000	0.038					1.00								
10731130	E	1.000	0.010													
10731150	E	1.000	0.010													
10851410	E	1.000	0.010													
10851430	E	1.000	0.010													
10851900	E	1.000	0.016													
10851910	E	1.000	0.016													
11071130	E	1.016	0.016													
11071150	E	1.016	0.016													
11071930	E	1.016	0.016													
11071950	E	1.016	0.016													
11071970	E	1.030	0.029													
13108110	C	1.000	0.800			0.19		0.60				0.12				0.08
13108130	C	1.000	1.000			0.19		0.60				0.12				0.08
13108150	C	1.000	0.500			0.19		0.60				0.12				0.08
13108210	C	1.000	0.900			0.19		0.60				0.12				0.08
13108250	C	1.000	0.900			0.19		0.60				0.12				0.08
13108320	C	1.000	0.500					1.00								
13108333	C	1.000	0.500			0.19		0.60				0.12				0.08
13108336	C	1.000	0.500			0.19		0.60				0.12				0.08
13108340	C	1.000	0.500			0.19		0.60				0.12				0.08
13108380	C	1.000	0.500			0.19		0.60				0.12				0.08
13108390	C	1.000	0.500			0.19		0.60				0.12				0.08
131083Z0	C	1.000	0.500			0.19		0.60				0.12				0.08
13108410	C	1.000	0.500			0.19		0.60				0.12				0.08
13108430	C	1.000	0.500			0.19		0.60				0.12				0.08
13108510	C	1.000	0.500			0.19		0.60				0.12				0.08
13108550	C	1.000	0.500			0.19		0.60				0.12				0.08
13203130	C	0.250	1.000			0.19		0.60				0.12				0.08
13203150	C	0.250	1.000			0.19		0.60				0.12				0.08
13203170	C	0.250	1.000			0.19		0.60				0.12				0.08
13203210	C	0.250	0.900			0.19		0.60				0.12				0.08
13203220	C	0.250	0.500			0.19		0.60				0.12				0.08
13203230	C	0.250	0.500			0.19		0.60				0.12				0.08
13203240	C	0.250	0.500			0.19		0.60				0.12				0.08
13203250	C	0.250	0.500			0.19		0.60				0.12				0.08

Code	Stage of supply chain	Unit conversion coefficient	Plastics mass fraction	Mass fraction of each polymer												
				PE-LD	PE-HD/MD	PP	PVC	PET	PS	EPS	ABS	PA	PC	PMMA	PUR	O
13203290	C	0.250	0.500			0.19		0.60				0.12				0.08
13203330	C	0.250	0.900			0.19		0.60				0.12				0.08
13203350	C	0.250	0.900			0.19		0.60				0.12				0.08
13204100	C	0.250	0.300			0.19		0.60				0.12				0.08
13911920	C	0.000	1.000			0.19		0.60				0.12				0.08
13921150	E	0.500	0.800		0.10	0.20		0.70								
13921190	E	0.500	0.200		0.10	0.20		0.70								
13921270	E	1.000	0.100					1.00								
13921359	E	1.000	0.800				0.10	0.90								
13921370	E	1.000	0.200				0.10	0.90								
13922150	E	1.000	1.000	0.30	0.30	0.40										
13922170	E	1.000	1.000	0.30	0.30	0.40										
13922173	E	1.000	1.000	0.30	0.30	0.40										
13922175	E	1.000	1.000	0.30	0.30	0.40										
13922190	E	1.000	1.000				1.00									
13922210	E	1.000	0.900		1.00											
13922230	E	1.000	0.900					1.00								
13922250	E	1.000	0.800		0.50			0.50								
13922270	E	1.000	0.600				1.00									
13922300	E	1.000	0.600									1.00				
13922430	E	2.000	0.800					0.70				0.30				
13941155	E	1.000	1.000	0.30	0.30	0.40										
13941160	E	1.000	1.000	0.10	0.10	0.50		0.20				0.10				
13941170	E	1.000	1.000	0.10	0.10	0.50		0.20				0.10				
13941233	E	1.000	1.000									1.00				
13941235	E	1.000	1.000									1.00				
13941253	E	1.000	1.000									1.00				
13941255	E	1.000	1.000									1.00				
14132110	E	1.000	0.700					1.00								
14132115	E	2.000	0.700					0.90				0.10				
14133110	E	1.000	0.700					1.00								
14133115	E	2.000	0.700					1.00								
15121210	E	5.000	0.500													
15121230	E	0.000	0.000													
15121250	E	0.000	0.000													
15201100	E	2.000	0.600													1.00
15201210	E	0.500	0.600													1.00
15201231	E	1.000	0.600													1.00
15201237	E	0.300	0.900													1.00
15201370	E	0.300	0.900													1.00
15201445	E	1.000	0.500													1.00

Code	Stage of supply chain	Unit conversion coefficient	Plastics mass fraction	Mass fraction of each polymer												
				PE-LD	PE-HD/MD	PP	PVC	PET	PS	EPS	ABS	PA	PC	PMMA	PUR	O
15202100	E	0.800	0.800													1.00
15203120	E	1.000	0.400													1.00
15203150	E	1.000	0.600													1.00
15204050	C	0.000	0.500													1.00
15204080	P	0.000	0.500													1.00
20161035	P	1.000	1.000	1.00												
20161039	P	1.000	1.000	1.00												
20161050	P	1.000	1.000		1.00											
20161070	P	1.000	1.000												1.00	1.00
20161090	P	1.000	1.000												1.00	1.00
20162035	P	1.000	1.000							1.00						
20162039	P	1.000	1.000						1.00							
20162050	P	1.000	1.000												1.00	1.00
20162070	P	1.000	1.000								1.00					
20162090	P	1.000	1.000												1.00	1.00
20163010	P	1.000	1.000				1.00									
20163023	P	1.000	1.000				1.00									
20163025	P	1.000	1.000				1.00									
20163040	P	1.000	1.000												1.00	1.00
20163060	P	1.000	1.000												1.00	1.00
20163090	P	1.000	1.000												1.00	1.00
20164013	P	1.000	1.000												1.00	1.00
20164015	P	1.000	1.000												1.00	1.00
20164020	P	1.000	1.000												1.00	1.00
20164030	P	1.000	1.000												1.00	1.00
20164040	P	1.000	1.000										1.00			
20164050	P	1.000	1.000												1.00	1.00
20164062	P	1.000	1.000					1.00								
20164064	P	1.000	1.000					1.00								
20164070	P	1.000	1.000													1.00
20164080	P	1.000	1.000													1.00
20164090	P	1.000	1.000													1.00
20165130	P	1.000	1.000			1.00										
20165150	P	1.000	1.000												1.00	1.00
20165230	P	1.000	1.000												1.00	1.00
20165250	P	1.000	1.000												1.00	1.00
20165270	P	1.000	1.000												1.00	1.00
20165350	P	1.000	1.000											1.00		
20165390	P	1.000	1.000												1.00	1.00
20165450	P	1.000	1.000									1.00				
20165490	P	1.000	1.000									1.00				

Code	Stage of supply chain	Unit conversion coefficient	Plastics mass fraction	Mass fraction of each polymer												
				PE-LD	PE-HD/MD	PP	PVC	PET	PS	EPS	ABS	PA	PC	PMMA	PUR	O
20165550	P	1.000	1.000													1.00
20165570	P	1.000	1.000													1.00
20165630	P	1.000	1.000													1.00
20165650	P	1.000	1.000													1.00
20165670	P	1.000	1.000													
20165700	P	1.000	1.000													1.00
20165920	P	1.000	1.000													1.00
20165940	P	1.000	1.000													1.00
20165960	P	1.000	1.000													1.00
20165970	P	1.000	1.000													1.00
22211050	C	1.000	1.000	0.50	0.50											
22211070	C	1.000	1.000				1.00									
22211090	C	1.000	1.000						0.25		0.25		0.25	0.25		
22212130	C	1.000	1.000												1.00	1.00
22212153	C	1.000	1.000		1.00											
22212155	C	1.000	1.000			1.00										
22212157	C	1.000	1.000				1.00									
22212170	C	1.000	1.000										0.50	0.50		
22212920	C	1.000	1.000									1.00				
22212935	C	1.000	1.000	0.20	0.40		0.20									
22212937	C	1.000	1.000	0.20	0.40		0.20									
22212950	C	1.000	1.000	0.20	0.40		0.20									
22212970	C	1.000	1.000				1.00									
22213010	C	1.000	1.000		1.00											
22213017	C	1.000	1.000		1.00											
22213021	C	1.000	1.000			1.00										
22213023	C	1.000	1.000			1.00										
22213025	C	1.000	1.000			1.00										
22213026	C	1.000	1.000			1.00										
22213029	C	1.000	1.000												1.00	1.00
22213030	C	1.000	1.000						1.00							
22213035	C	1.000	1.000				1.00									
22213036	C	1.000	1.000				1.00									
22213037	C	1.000	1.000				1.00									
22213038	C	1.000	1.000				1.00									
22213053	C	1.000	1.000											1.00		
22213059	C	1.000	1.000												1.00	1.00
22213061	C	1.000	1.000										1.00			
22213063	C	1.000	1.000													1.00
22213065	C	1.000	1.000					1.00								
22213067	C	1.000	1.000					1.00								

Code	Stage of supply chain	Unit conversion coefficient	Plastics mass fraction	Mass fraction of each polymer												
				PE-LD	PE-HD/MD	PP	PVC	PET	PS	EPS	ABS	PA	PC	PMMA	PUR	O
22213069	C	1.000	1.000					0.33					0.33			0.33
22213070	C	1.000	1.000												1.00	1.00
22213082	C	1.000	1.000									1.00				
22213086	C	1.000	1.000												1.00	1.00
22213090	C	1.000	1.000												1.00	1.00
22214120	C	1.000	1.000						1.00							
22214130	C	1.000	1.000				1.00									
22214150	C	1.000	1.000													
22214170	C	1.000	1.000												1.00	1.00
22214180	C	1.000	1.000		0.50											0.50
22214230	C	1.000	1.000													1.00
22214250	C	1.000	1.000													1.00
22214275	C	1.000	1.000													1.00
22214279	C	1.000	1.000													1.00
22214280	C	1.000	1.000													1.00
22221100	E	1.000	1.000	1.00												
22221200	E	1.000	1.000			0.60	0.40									
22221300	E	1.000	1.000													
22221450	E	0.016	1.000													
22221470	E	0.050	1.000													
22221910	E	1.000	1.000													
22221920	E	1.000	1.000	0.30	0.30	0.40										
22221925	E	1.000	1.000	0.30	0.30	0.40										
22221930	E	1.000	1.000	0.30	0.30	0.40										
22221940	E	1.000	1.000									1.00				
22221950	E	1.000	1.000													
22221990	E	0.500	0.800													
22231155	E	2.500	0.400				1.00									
22231159	E	2.500	1.000				1.00									
22231190	E	2.500	0.700													0.50
22231250	E	1.500	0.700			1.00										
22231270	E	1.000	0.900			1.00										
22231290	E	3.000	0.700			1.00										
22231300	E	1.000	0.900	0.50	0.50											
22231450	E	20.000	0.800				1.00									
22231470	E	1.000	0.900				1.00									
22231500	E	2.500	0.800				1.00									
22231950	C	1.000	0.100				1.00									
22231990	C	1.000	0.100				1.00									
22232000	E	1.000	0.700				1.00									
22291000	E	1.000	0.900			0.19		0.60				0.12				0.08

				Mass fraction of each polymer												
Code	Stage of supply chain	Unit conversion coefficient	Plastics mass fraction	PE-LD	PE-HD/MD	PP	PVC	PET	PS	EPS	ABS	PA	PC	PMMA	PUR	O
22292130	C	1.000	1.000			1.00										
22292140	C	1.000	1.000			1.00										
22292150	C	1.000	1.000			1.00										
22292170	C	1.000	1.000			1.00										
22292190	C	1.000	1.000			1.00										
22292230	C	1.000	1.000			1.00										
22292240	C	1.000	1.000			1.00										
22292250	C	1.000	1.000			1.00										
22292270	C	1.000	1.000			1.00										
22292290	C	1.000	1.000			1.00										
22292320	E	1.000	1.000													
22292340	E	1.000	1.000													
22292350	E	1.000	0.000													
22292390	E	1.000	0.800													
22292400	C	1.000	0.200										1.00			
22292500	E	1.000	1.000			1.00										
22292610	C	1.000	1.000				1.00									
22292620	E	1.000	0.900													
22292630	E	1.000	1.000													
22292910	E	1.000	0.500													
22292915	E	1.000	1.000													
22292920	E	0.300	1.000						1.00							
22292950	E	1.000	1.000													
22292990	E	1.000	1.000													
22292995	E	1.000	1.000													
22299110	C	1.000	0.800													
22299125	C	1.000	0.800													
22299127	C	1.000	0.600													
22299130	C	1.000	0.600													
22299140	C	1.000	0.800													
22299150	C	1.000	0.800													
22299160	C	1.000	0.800													
22299180	C	1.000	0.800													
22299193	C	1.000	0.800													
22299197	C	1.000	0.800													
26113003	E	0.250	0.300													
26113006	E	0.250	0.300													
26113023	E	0.250	0.300													
26113027	E	0.100	0.300													
26113034	E	0.001	0.300													
26113054	E	0.001	0.300													

				Mass fraction of each polymer												
Code	Stage of supply chain	Unit conversion coefficient	Plastics mass fraction	PE-LD	PE-HD/MD	PP	PVC	PET	PS	EPS	ABS	PA	PC	PMMA	PUR	O
26113065	E	0.001	0.300													
26113067	E	0.001	0.300													
26113080	E	0.001	0.300													
26113091	E	0.100	0.300													
26113094	E	0.001	0.300													
26114010	E	0.100	0.200													
26114090	C	0.000	0.300													
26115020	E	0.200	0.400													
26115050	E	0.200	0.400													
26121020	E	0.200	0.400													
26121050	E	0.200	0.400													
26123000	E	0.010	0.300													
26201100	E	3.000	0.400													
26201200	E	100.000	0.200													
26201300	E	6.000	0.300													
26201400	E	3.000	0.300													
26201500	E	3.000	0.300													
26201640	E	5.000	0.300													
26201650	E	0.500	0.300													
26201660	E	0.500	0.300													
26201700	E	4.000	0.300													
26201800	E	5.000	0.300													
26202100	E	1.000	0.200													
26301300	E	3.000	0.300													
26302200	E	0.200	0.050													
26302330	E	0.400	0.300													
26302370	E	0.400	0.300													
26401100	E	0.500	0.300													
26401250	E	0.500	0.300													
26401270	E	1.000	0.300													
26401290	E	1.000	0.300													
26402020	E	3.000	0.300													
26402040	E	15.000	0.300													
26402090	E	15.000	0.300													
26403300	E	5.000	0.300													
26403400	E	5.000	0.300													
26403420	E	5.000	0.300													
26403460	E	15.000	0.300													
26404100	E	0.100	0.300													
26404235	E	0.500	0.300													
26404237	E	2.500	0.300													

Code	Stage of supply chain	Unit conversion coefficient	Plastics mass fraction	Mass fraction of each polymer												
				PE-LD	PE-HD/MD	PP	PVC	PET	PS	EPS	ABS	PA	PC	PMMA	PUR	O
26404239	E	2.500	0.200													
26404270	E	0.050	0.400													
26406000	E	3.000	0.300													
26406050	E	3.000	0.300													
26511180	E	0.500	0.300													
26511200	E	1.000	0.300													
26511215	E	1.000	0.300													
26511235	E	1.000	0.300													
26511239	E	1.000	0.300													
26511250	E	1.000	0.300													
26511270	E	1.000	0.300													
26511280	E	3.000	0.300													
26514400	E	0.500	0.300													
26514500	E	0.500	0.300													
26514520	E	0.500	0.300													
26514530	E	0.500	0.300													
26514555	E	0.500	0.300													
26515135	E	0.500	0.300													
26515139	E	0.100	0.600													
26515150	E	0.200	0.600													
26515175	E	0.100	0.600													
26515235	E	0.200	0.600													
26515239	E	0.100	0.600													
26515271	E	0.100	0.600													
26515279	E	0.100	0.300													
26515283	E	0.100	0.600													
26515313	E	0.200	0.300													
26515383	E	0.200	0.300													
26516210	E	1.000	0.300													
26516255	E	1.000	0.300													
26516370	E	1.000	0.300													
26516650	E	0.500	0.300													
26516670	E	1.000	0.300													
26517015	E	0.100	0.500													
26517030	E	0.100	0.300													
26517090	E	0.500	0.300													
26701300	E	0.500	0.300													
27123203	E	3.000	0.800													
27123205	E	5.000	0.800													
27124030	E	2.000	0.800													
27331310	E	0.300	0.800													

Code	Stage of supply chain	Unit conversion coefficient	Plastics mass fraction	Mass fraction of each polymer												
				PE-LD	PE-HD/MD	PP	PVC	PET	PS	EPS	ABS	PA	PC	PMMA	PUR	O
27331330	E	0.300	0.800													
27331350	E	0.300	0.800													
27331410	E	1.000	0.800													
27331430	E	1.000	0.800													
27511110	E	135.000	0.200													
27511133	E	100.000	0.200													
27511135	E	100.000	0.200													
27511150	E	100.000	0.200													
27511170	E	100.000	0.200													
27511200	E	70.000	0.200													
27511300	E	95.000	0.100													
27511400	E	2.000	0.200													
27511530	E	15.000	0.400													
27511580	E	15.000	0.400													
27512123	E	5.000	0.300													
27512125	E	5.000	0.300													
27512170	E	1.000	0.300													
27512190	E	1.000	0.300													
27512200	E	0.200	0.300													
27512310	E	0.800	0.300													
27512313	E	0.200	0.300													
27512315	E	0.100	0.300													
27512330	E	0.100	0.300													
27512350	E	0.100	0.300													
27512370	E	1.000	0.300													
27512410	E	3.000	0.300													
27512430	E	3.000	0.300													
27512450	E	1.500	0.300													
27512490	E	5.000	0.300													
27512530	E	1.000	0.300													
27512700	E	5.000	0.300													
28231000	E	0.100	0.300													
28231100	E	5.000	0.200													
28231200	E	0.050	0.400													
28231300	E	2.000	0.300													
28232100	E	100.000	0.300													
28232110	E	50.000	0.300													
28232200	E	5.000	0.300													
28232300	E	5.000	0.300													
28241113	E	5.000	0.200													
28241115	E	5.000	0.050													

Code	Stage of supply chain	Unit conversion coefficient	Plastics mass fraction	Mass fraction of each polymer												
				PE-LD	PE-HD/MD	PP	PVC	PET	PS	EPS	ABS	PA	PC	PMMA	PUR	O
28241117	E	5.000	0.050													
28241120	E	5.000	0.050													
28251220	E	40.000	0.300													
28251240	E	20.000	0.300													
28251250	E	40.000	0.300													
28251270	E	10.000	0.300													
28251410	E	5.000	0.300													
28251420	E	5.000	0.300													
28251430	E	5.000	0.300													
28252010	E	0.100	0.300													
28252030	E	3.000	0.300													
28252050	E	3.000	0.300													
28252070	E	3.000	0.300													
28293200	E	2.000	0.200													
28294330	E	150.000	0.200													
28294350	E	150.000	0.200													
28942230	E	130.000	0.200													
28942270	E	100.000	0.200													
29102100	E	1200.000	0.100													
29102230	E	2500.000	0.100													
29102250	E	2000.000	0.100													
29102310	E	1200.000	0.100													
29102330	E	1500.000	0.100													
29102340	E	2500.000	0.100													
29102353	E	2000.000	0.100													
29102355	E	2500.000	0.100													
29102400	E	2000.000	0.100													
29102410	E	1500.000	0.100													
29102430	E	1500.000	0.100													
29102450	E	1700.000	0.100													
29102490	E	2000.000	0.100													
29103000	E	12500.000	0.100													
29104110	E	3000.000	0.100													
29104130	E	12500.000	0.070													
29104140	E	30000.000	0.050													
29104200	E	12500.000	0.070													
29104300	E	8000.000	0.050													
29105200	E	250.000	0.100													
29202210	E	600.000	0.100													
29202230	E	2000.000	0.100													
29202250	E	4000.000	0.100													

Code	Stage of supply chain	Unit conversion coefficient	Plastics mass fraction	Mass fraction of each polymer												
				PE-LD	PE-HD/MD	PP	PVC	PET	PS	EPS	ABS	PA	PC	PMMA	PUR	O
29202292	E	1300.000	0.100													
29202298	E	2000.000	0.100													
29202300	E	1600.000	0.100													
29322030	E	0.300	0.500									1.00				
30121970	E	90.000	0.700													
30202000	E	45000.000	0.050													
30203200	E	45000.000	0.050													
31002090	E	0.000	0.500			1.00										
31031250	E	30.000	0.600					1.00								
31091430	E	30.000	0.800			1.00										
32301530	E	0.000	0.300												1.00	1.00
32301550	E	0.000	0.600												1.00	1.00
32301560	E	0.200	0.500												1.00	1.00
32301580	E	0.100	0.500													1.00
32301590	E	0.000	0.500												1.00	1.00
32301600	E	0.000	0.800												1.00	1.00
32401100	E	2.000	0.800	0.50							0.50					
32401200	E	1.000	0.800	0.50							0.50					
32401300	E	0.000	0.800	0.50							0.50					
32402000	E	0.000	0.800	0.50							0.50					
32403100	E	1.000	0.800								1.00					
32403200	E	2.000	0.500								1.00					
32403900	E	0.000	0.500								1.00					
32403920	E	2.000	0.400								1.00					
32403940	E	0.000	0.800								1.00					
32403960	E	0.000	0.200								1.00					
32403990	E	0.000	0.500								1.00					
32404100	E	1.000	0.300													
32404210	E	0.000	0.300													
32404230	E	2.000	0.300													
32404250	E	2.000	0.300													
32404270	E	1.000	0.300													
32504350	E	2.000	0.700													
32911110	E	0.500	0.100													
32911150	E	0.500	0.500													
32911190	E	0.500	0.500													
32911210	E	0.200	0.600													
32911235	E	0.200	0.600													
32911237	E	0.200	0.500													
32911930	E	0.100	0.400													
32991130	E	0.100	0.500												1.00	1.00

Code	Stage of supply chain	Unit conversion coefficient	Plastics mass fraction	Mass fraction of each polymer												
				PE-LD	PE-HD/MD	PP	PVC	PET	PS	EPS	ABS	PA	PC	PMMA	PUR	O
32991150	E	1.000	0.600		0.30						0.40		0.30			
32991190	E	1.000	0.600													1.00
32991210	E	0.050	0.600			1.00										
32991230	E	0.050	0.600			1.00										
32991350	E	0.100	0.400			1.00										
32991430	E	0.010	0.300			1.00										
32994110	E	0.100	0.300								1.00					
399900Z0	E	0.000	0.300				1.00									

Table S 2 details the allocation of PRODCOM (Eurostat, 2018) product codes by end-use applications. This was used to allocate codes to end-use applications for all codes classified as 'stage of supply chain' = 'E' in Table S 1, and for all other if that information was either obvious or available from the literature (PlasticsEurope, 2019). Each PRODCOM code would have at least detailed allocation by polymer or by end-use application. This table can be accessed in xlsx format online at https://doi.org/10.17863/CAM.92501

Table S 2. Classification of each code of PRODCOM product categories by end-use application. Values are mass shares for each PROCOM code.

Code	Mass fraction of each end-use application								
	Packaging	Construction	Vehicles	Consumer products: EEE	Consumer products: other	Consumer products: personal care	Textiles	Agriculture	Others
10511133	1								
10511137	1								
10511142	1								
10511148	1								
10511210	1								
10511220	1								
10511230	1								
10511240	1								
10512230	1								
10512260	1								
10515245	1								
10731130	1								
10731150	1								
10851410	1								
10851430	1								
10851900	1								

	Mass fraction of each end-use application								
Code	Packaging	Construction	Vehicles	Consumer products: EEE	Consumer products: other	Consumer products: personal care	Textiles	Agriculture	Others
10851910	1								
11071130	1								
11071150	1								
11071930	1								
11071950	1								
11071970	1								
13108110							1		
13108130							1		
13108150							1		
13108210							1		
13108250							1		
13108320							1		
13108333							1		
13108336							1		
13108340							1		
13108380							1		
13108390							1		
131083Z0							1		
13108410							1		
13108430							1		
13108510							1		
13108550							1		
13203130							1		
13203150							1		
13203170							1		
13203210							1		
13203220							1		
13203230							1		
13203240							1		
13203250							1		
13203290							1		
13203330							1		
13203350							1		
13204100							1		
13911920							1		
13921150							1		
13921190							1		
13921270							1		
13921359							1		
13921370							1		
13922150	1								

Code	Mass fraction of each end-use application								
	Packaging	Construction	Vehicles	Consumer products: EEE	Consumer products: other	Consumer products: personal care	Textiles	Agriculture	Others
13922170	1								
13922173	1								
13922175	1								
13922190	1								
13922210							1		
13922230							1		
13922250							1		
13922270							1		
13922300							1		
13922430							1		
13941155								1	
13941160								1	
13941170								1	
13941233								1	
13941235								1	
13941253								1	
13941255								1	
14132110							1		
14132115							1		
14133110							1		
14133115							1		
15121210					1				
15121230					1				
15121250					1				
15201100					1				
15201210					1				
15201231					1				
15201237					1				
15201370					1				
15201445					1				
15202100					1				
15203120					1				
15203150					1				
15204050					1				
15204080					1				
20161035									
20161039									
20161050									
20161070									
20161090									
20162035									

Code	Mass fraction of each end-use application									
	Packaging	Construction	Vehicles	Consumer products: EEE	Consumer products: other	Consumer products: personal care	Textiles	Agriculture	Others
20162039									
20162050									
20162070									
20162090									
20163010									
20163023									
20163025									
20163040									
20163060									
20163090									
20164013									
20164015									
20164020									
20164030									
20164040									
20164050									
20164062									
20164064									
20164070									
20164080									
20164090									
20165130									
20165150									
20165230									
20165250									
20165270									
20165350									
20165390									
20165450									
20165490									
20165550									
20165570									
20165630									
20165650									
20165670									
20165700									
20165920									
20165940									
20165960									
20165970									
22211050									

Code	Mass fraction of each end-use application								
	Packaging	Construction	Vehicles	Consumer products: EEE	Consumer products: other	Consumer products: personal care	Textiles	Agriculture	Others
22211070									
22211090									
22212130		1							
22212153									
22212155									
22212157									
22212170									
22212920									
22212935									
22212937									
22212950									
22212970									
22213010									
22213017									
22213021									
22213023									
22213025									
22213026									
22213029									
22213030									
22213035									
22213036									
22213037									
22213038									
22213053									
22213059									
22213061									
22213063									
22213065									
22213067									
22213069									
22213070									
22213082									
22213086									
22213090									
22214120									
22214130									
22214150									
22214170									
22214180									
22214230									

Code	Mass fraction of each end-use application								
	Packaging	Construction	Vehicles	Consumer products: EEE	Consumer products: other	Consumer products: personal care	Textiles	Agriculture	Others
22214250									
22214275									
22214279									
22214280									
22221100	1								
22221200	1								
22221300	1								
22221450	1								
22221470	1								
22221910					1				
22221920	1								
22221925					1				
22221930					1				
22221940					1				
22221950	1								
22221990	1								
22231155		1							
22231159		1							
22231190		1							
22231250		1							
22231270		1							
22231290		1							
22231300		1							
22231450		1							
22231470		1							
22231500		1							
22231950		1							
22231990		1							
22232000		1							
22291000							1		
22292130									
22292140									
22292150									
22292170									
22292190									
22292230									
22292240									
22292250									
22292270									
22292290									
22292320					1				

Code	Mass fraction of each end-use application								
	Packaging	Construction	Vehicles	Consumer products: EEE	Consumer products: other	Consumer products: personal care	Textiles	Agriculture	Others
22292340					1				
22292350					1				
22292390					1				
22292400				1					
22292500					1				
22292610		1							
22292620					1				
22292630					0.5			0.5	
22292910						1			
22292915						1			
22292920					1				
22292950	0.418	0.200	0.095	0.056	0.030	0.026	0.000	0.012	0.163
22292990	0.418	0.200	0.095	0.056	0.030	0.026	0.000	0.012	0.163
22292995	0.418	0.200	0.095	0.056	0.030	0.026	0.000	0.012	0.163
22299110					1				
22299125					1				
22299127				1					
22299130				1					
22299140				1					
22299150			1						
22299160			1						
22299180			1						
22299193				1					
22299197				1					
26113003				1					
26113006				1					
26113023				1					
26113027				1					
26113034				1					
26113054				1					
26113065				1					
26113067				1					
26113080				1					
26113091				1					
26113094				1					
26114010				1					
26114090				1					
26115020				1					
26115050				1					
26121020				1					
26121050				1					

	Mass fraction of each end-use application								
Code	Packaging	Construction	Vehicles	Consumer products: EEE	Consumer products: other	Consumer products: personal care	Textiles	Agriculture	Others
26123000				1					
26201100				1					
26201200				1					
26201300				1					
26201400				1					
26201500				1					
26201640				1					
26201650				1					
26201660				1					
26201700				1					
26201800				1					
26202100				1					
26301300				1					
26302200				1					
26302330				1					
26302370				1					
26401100				1					
26401250				1					
26401270				1					
26401290				1					
26402020				1					
26402040				1					
26402090				1					
26403300				1					
26403400				1					
26403420				1					
26403460				1					
26404100				1					
26404235				1					
26404237				1					
26404239				1					
26404270				1					
26406000				1					
26406050				1					
26511180				1					
26511200				1					
26511215				1					
26511235				1					
26511239				1					
26511250				1					
26511270				1					

Code	Mass fraction of each end-use application: Packaging	Construction	Vehicles	Consumer products: EEE	Consumer products: other	Consumer products: personal care	Textiles	Agriculture	Others
26511280				1					
26514400				1					
26514500				1					
26514520				1					
26514530				1					
26514555				1					
26515135				1					
26515139				1					
26515150				1					
26515175				1					
26515235				1					
26515239				1					
26515271				1					
26515279				1					
26515283				1					
26515313				1					
26515383				1					
26516210				1					
26516255				1					
26516370				1					
26516650				1					
26516670				1					
26517015				1					
26517030				1					
26517090				1					
26701300				1					
27123203				1					
27123205				1					
27124030				1					
27331310				1					
27331330				1					
27331350				1					
27331410				1					
27331430				1					
27511110				1					
27511133				1					
27511135				1					
27511150				1					
27511170				1					
27511200				1					
27511300				1					

Code	Mass fraction of each end-use application								
	Packaging	Construction	Vehicles	Consumer products: EEE	Consumer products: other	Consumer products: personal care	Textiles	Agriculture	Others
27511400				1					
27511530				1					
27511580				1					
27512123				1					
27512125				1					
27512170				1					
27512190				1					
27512200				1					
27512310				1					
27512313				1					
27512315				1					
27512330				1					
27512350				1					
27512370				1					
27512410				1					
27512430				1					
27512450				1					
27512490				1					
27512530				1					
27512700				1					
28231000				1					
28231100				1					
28231200				1					
28231300				1					
28232100				1					
28232110				1					
28232200				1					
28232300				1					
28241113				1					
28241115				1					
28241117				1					
28241120				1					
28251220				1					
28251240				1					
28251250				1					
28251270				1					
28251410				1					
28251420				1					
28251430				1					
28252010				1					
28252030				1					

Code	Mass fraction of each end-use application								
	Packaging	Construction	Vehicles	Consumer products: EEE	Consumer products: other	Consumer products: personal care	Textiles	Agriculture	Others
28252050				1					
28252070				1					
28293200				1					
28294330				1					
28294350				1					
28942230				1					
28942270				1					
29102100			1						
29102230			1						
29102250			1						
29102310			1						
29102330			1						
29102340			1						
29102353			1						
29102355			1						
29102400			1						
29102410			1						
29102430			1						
29102450			1						
29102490			1						
29103000			1						
29104110			1						
29104130			1						
29104140			1						
29104200			1						
29104300			1						
29105200			1						
29202210			1						
29202230			1						
29202250			1						
29202292			1						
29202298			1						
29202300			1						
29322030			1						
30121970			1						
30202000			1						
30203200			1						
31002090					1				
31031250					1				
31091430					1				
32301530					1				

Code	Mass fraction of each end-use application								
	Packaging	Construction	Vehicles	Consumer products: EEE	Consumer products: other	Consumer products: personal care	Textiles	Agriculture	Others
32301550					1				
32301560					1				
32301580					1				
32301590					1				
32301600								1	
32401100					1				
32401200					1				
32401300					1				
32402000					1				
32403100					1				
32403200					1				
32403900					1				
32403920					1				
32403940					1				
32403960					1				
32403990					1				
32404100					1				
32404210					1				
32404230					1				
32404250					1				
32404270					1				
32504350					1				
32911110					1				
32911150					1				
32911190					1				
32911210					1				
32911235					1				
32911237					1				
32911930					1				
32991130					1				
32991150					1				
32991190					1				
32991210					1				
32991230					1				
32991350					1				
32991430					1				
32994110					1				
399900Z0		1							

Table S 3 shows the average polymer compositions for each end-use application for Europe, as reported by PlasticsEurope (PlasticsEurope, 2019).

Table S 3. Average polymer composition by end-use application. Adapted from PlasticsEurope (2019). Polymer acronyms: PE-LD= low density polyethylene, PE-HD/MD=high or medium density polyethylene, PP=polypropylene, PVC=polyvinyl chloride, PET=polyethylene terephthalate, PS=polystyrene, EPS=expanded polystyrene, ABS=acrylonitrile butadiene styrene, PA=polyamide, PC=polycarbonate, PMMA=poly(methyl methacrylate), PUR= polyurethane, O = other

	PE-LD	PE-HD/MD	PP	PVC	PET	PS	EPS	ABS	PA	PC	PMMA	PUR	Other
Packaging	30.1%	18.3%	23.3%	1.7%	18.3%	4.8%	1.2%	0.1%	0.4%	0.0%	0.0%	0.1%	1.6%
Construction	4.9%	13.3%	8.2%	38.3%	0.0%	1.6%	12.2%	0.1%	0.6%	1.6%	0.6%	9.1%	9.5%
Automotive	2.6%	6.4%	28.0%	2.6%	0.0%	1.3%	0.0%	5.3%	7.6%	1.3%	0.8%	15.3%	28.8%
Electric/Electronic	9.0%	4.4%	15.2%	2.3%	0.1%	5.8%	0.0%	9.0%	5.8%	9.0%	0.1%	9.0%	30.4%
Consumer products *	1.2%	10.7%	33.9%	8.5%	0.0%	10.7%	0.1%	4.8%	0.3%	2.1%	0.8%	1.2%	25.7%
Personal care *	1.2%	10.9%	34.5%	8.6%	0.0%	10.9%	0.1%	3.4%	0.3%	2.2%	0.5%	1.2%	26.1%
Agriculture	32.4%	0.2%	37.2%	8.1%	0.1%	1.5%	0.0%	0.0%	0.0%	0.0%	0.0%	0.0%	20.6%
Others	15.0%	6.1%	17.8%	3.1%	0.0%	3.1%	0.0%	3.7%	2.0%	1.1%	1.1%	20.9%	26.1%

* Reported by PlasticsEurope as "Household, Leisure and Sports".

2. End-of-life waste estimates

PlasticsEurope (2019) publishes estimates of plastic waste collected in the UK by type of end-of-life management option between 2006 and 2016. 2017 data on plastic waste recycling is provided by WRAP (2018), and the remaining values were estimated using the same shares of landfill/incineration/recycling as in 2016. This data is reproduced in Table S 4.

Table S 4. Plastic waste collection in the UK by management option.

Year	Landfill (Mt)	Incineration (Mt)	Recycling (Mt)	TOTAL (Mt)	Source
2006	2.590	0.231	0.525	3.346	(PlasticsEurope, 2019)
2007	2.8	0.2	0.6	3.6	
2008	2.7	0.2	0.7	3.6	
2009	2.7	0.2	0.7	3.6	
2010	2.6	0.3	0.8	3.7	
2011	2.9	0.2	0.7	3.9	
2012	2.9	0.3	0.9	4.1	
2013	2.2	0.6	1.0	3.8	
2014	1.6	1.0	1.1	3.6	
2015	1.3	1.3	1.2	3.8	
2016	1.116	1.441	1.210	3.767	

Year	Landfill (Mt)	Incineration (Mt)	Recycling (Mt)	TOTAL (Mt)	Source
2017	1.0	1.3	1.1	3.4	(WRAP, 2018; PlasticsEurope, 2019)

The recycling capacity in the UK is limited to around 400 kt per year (WRAP, 2019), and since the annual mass of plastic waste collected for recycling is consistently greater than UK domestic recycling capacity, the surplus is exported to be recycled in other countries. These flows can be estimated from the UK Trade Statistics (HMRC, 2019) on plastic waste exports. However, most of these destination countries have weak waste management systems that result in high rates of leakage to the land and marine ecosystems. These rates were estimated by Jambeck *et al.* (2015) for all countries to where the UK exports plastic waste. The results are summarised in Table S 5.

Table S 5. Destination of UK plastic waste send to recycling in 2017.

Waste destination		Mass flow (Mt)
UK		0.439
WRAP (2019)Exports	EU	0.134
	Turkey	0.041
	Other Europe	0.008
	SE Asia	0.199
	China	0.242
	India	0.020
	Other Asia	0.014
	Oceania	0.000005
	Latin America and Caribbean	0.000445
	Middle East and N Africa	0.0014
	North America	0.000761
	Sub-Saharan Africa	0.000063
Total UK waste for recycling		1.1

3. Recycling yields

WRAP (2018, 2019) report data on the end markets for packaging and non-packaging plastics in the UK. Figure S 1 to Figure S 5 show the destination of recycled plastics in the UK by polymer. This data was used to compute an estimate of recycling yield losses, which are summarised in Table S 6.

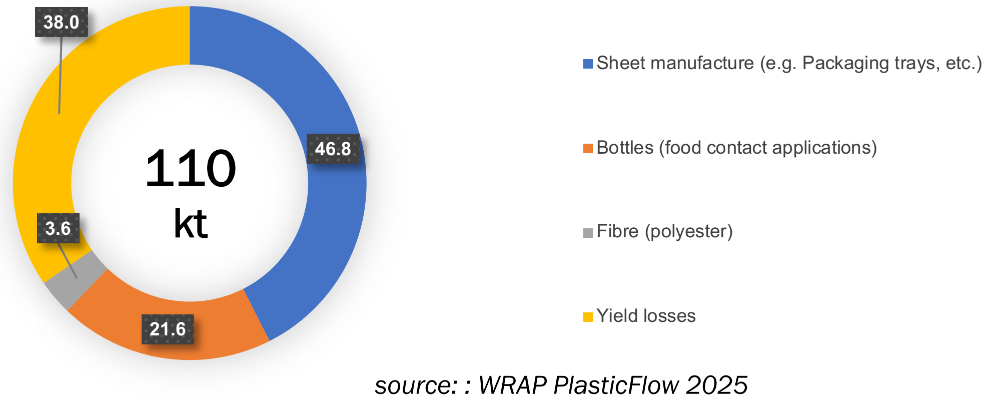

Figure S 1. End markets for recycled PET for the UK in 2017.

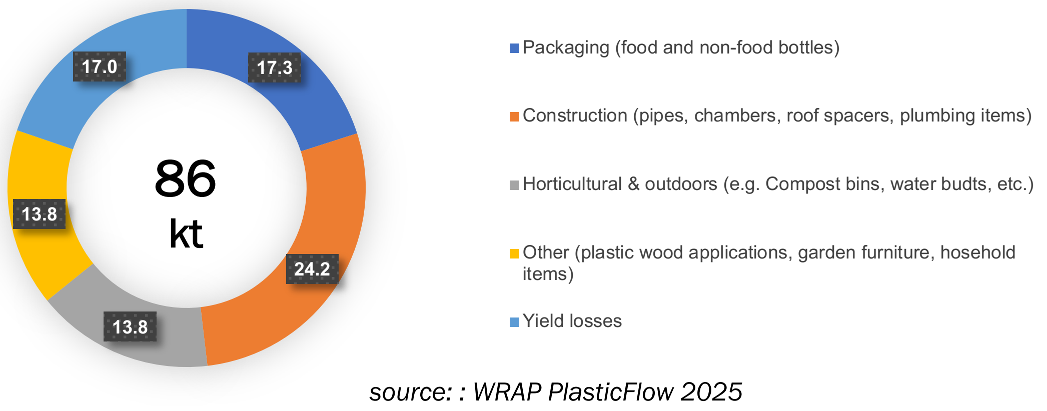

Figure S 2. End markets for recycled PE-HD for the UK in 2017.

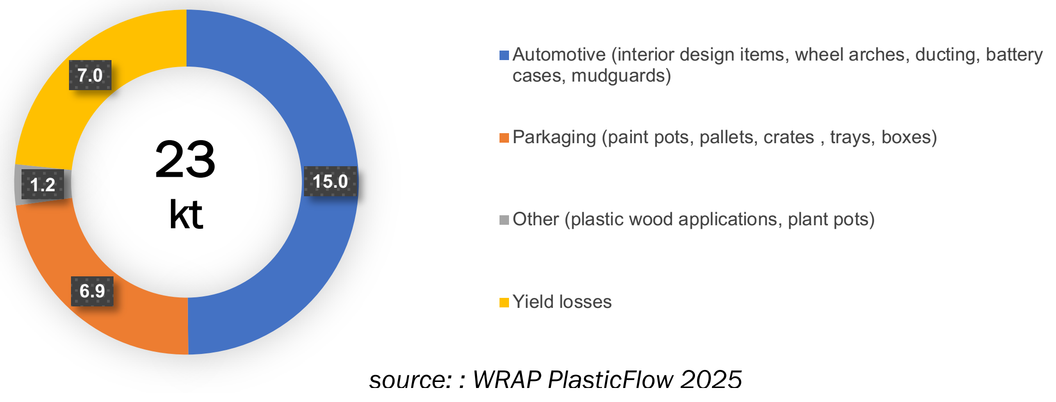

Figure S 3. End markets for recycled PP for the UK in 2017.

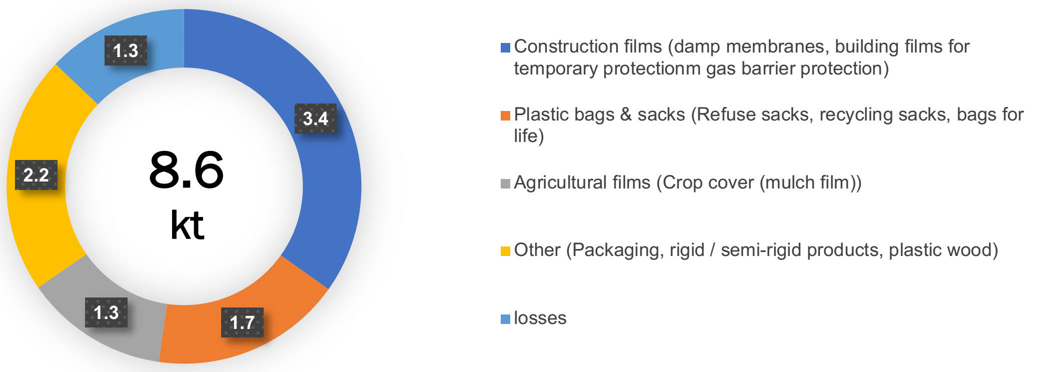

source: : WRAP PlasticFlow 2025

Figure S 4. End markets for recycled PE-LD for the UK in 2017.

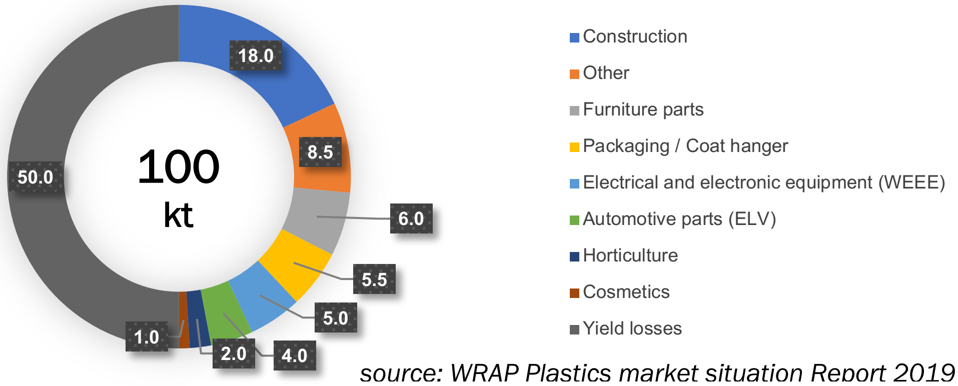

source: WRAP Plastics market situation Report 2019

Figure S 5. End markets for recycled WEEE and ELV plastics.

Table S 6. Estimated recycling yield losses for plastic polymers in the UK.

Polymer	Recycling yield losses	Source and notes
PET	34.6%	Source: WRAP (2018)
PE-HD	19.8%	Source: WRAP (2018)
PE-LD	12.9%	Source: WRAP (2018)
PP	23.3%	Source: WRAP (2018)
PVC	4%	Source: UK Plastic Recyclers
others	50%	Non-packaging waste is dominated by construction, vehicles, and consumer products. WRAP (2019) only reports end market data for WEEE and vehicles, but construction waste is dominated by PVC. Therefore, we

Polymer	Recycling yield losses	Source and notes
		assumed a yield loss for non-packaging polymers not listed above as being the same as for WEEE and vehicle plastics waste recycling.

4. Log-Normal parameter estimation

A log-normal distribution function was used to model the dynamics of plastic products in service. The log-normal probability distribution function ($f(x,S,M)$ in equation (1)) is a function of time (here interpreted as the age of end-use products made of plastics) and two parameters M and S. These can be estimated from the mean μ and standard deviation σ of product lifetimes, according to equation (2).

$$f(x,S,M) = \frac{1}{\sqrt{2\pi}Sx} e^{-\frac{(\ln x - M)^2}{2S^2}} \quad (1)$$

$$\begin{cases} \mu = e^{M+\frac{S^2}{2}} \\ \sigma = \sqrt{e^{S^2+2M}\left(e^{S^2}-1\right)} \end{cases} \quad (2)$$

For this analysis we used data estimated by Geyer *et al.* (2017) for the mean μ and standard deviation σ of various categories of product lifetimes.

The hazard rate of the log-normal distribution may be interpreted an instantaneous probability of failure at age t. This hazard function is then used in the stock model to calculate the share of plastic products of each category of age t that are disposed in any given year. This hazard function is defined as:

$$h(x,S,M) = \frac{\frac{1}{\sqrt{2\pi}Sx} e^{-\frac{(\ln x - M)^2}{2S^2}}}{1 - \Phi\left(\frac{\ln x - M}{S}\right)}, \quad (3)$$

where Φ is the cumulative distribution function of the standard normal $N(\mu = 0, \sigma^2 = 1)$. For each year n, the mass of plastics of each product category of age t that is sent to waste ($B_{out,n,t}$) is therefore calculate according to equation (4).

$$B_{out,n,t} = S_{n-1,t} \frac{\frac{1}{\sqrt{2\pi}Sx} e^{-\frac{(\ln x - M)^2}{2S^2}}}{1 - \Phi\left(\frac{\ln x - M}{S}\right)} \quad (4)$$

5. Demand estimates

The dynamic model described in the main text requires an estimation of a required amount of stock of plastics in service (S_n) for each year n and product category. Figure S 6 summarises the relations between all variables in the model.

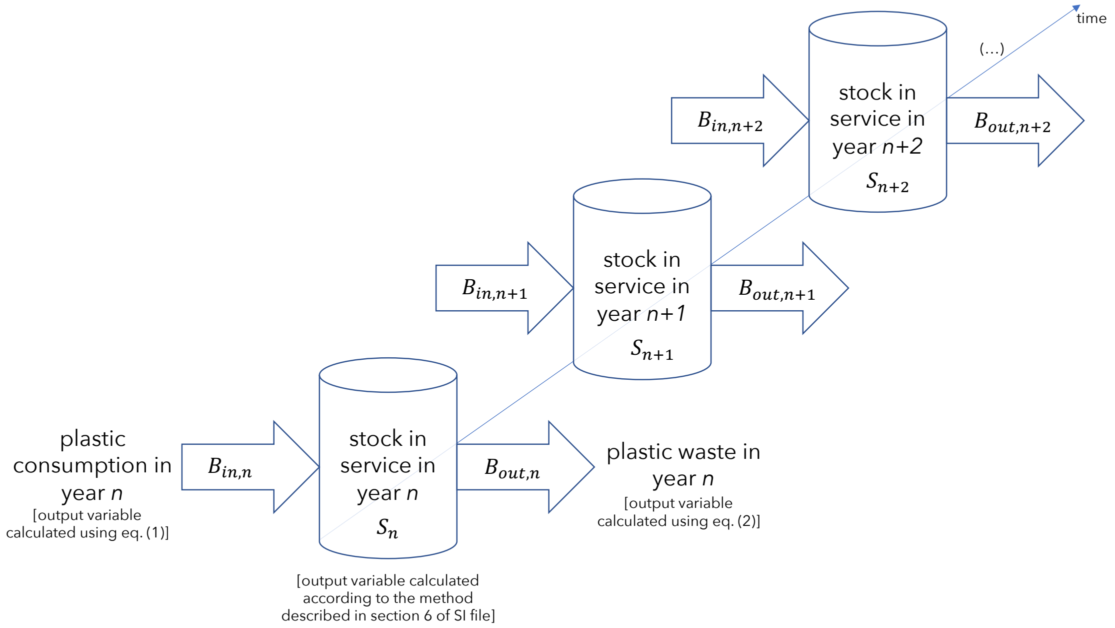

Figure S 6. Schematic representation of the dynamic model and key variable inputs and outputs.

Table S 7 shows the stocks per capita used in this analysis, which were multiplied by the UK population projections produced by the ONS (2017) to obtain the total mass of plastics in service required for each year (S_n). The stocks per capita shown in Table S 7 are a model output and resulted from the estimation of the accumulation of plastics in use, using our mapping methodology based on PRODCOM trade flows since 1995. We provide the detailed analysis of stock estimates below.

Table S 7. Estimated stock per capita by product category in the UK.

Product category	Stock per capita (kg per capita)	Notes
Agriculture	10.0	Average of the past 5 years.
Vehicles	130.2	Average of the past 5 years.
Construction		(see below)
Consumer products	22.0	Average of the past 5 years.
Electric	74.6	Average of the past 5 years.
Personal care	0.06	Average of the past 5 years.
Textiles	11.0	Average of the past 5 years.
Packaging	26.4	Average of the past 5 years.
Others	52.8	Average of the past 5 years.

UK trade statistics are available since 1995. This raw data could then be used to estimate the annual flows of plastic added to the stock in service for every year since 1995. This gives us a long-enough time series to compute current stocks in service, since the average lifetime of all plastic products (except construction) is much shorter than the length of the time series since 1995. Therefore, we were able to apply the Weibull distribution functions described in section 4 to compute the waste generation for each product category and age. Using this procedure, we observed that for recent years, plastic stocks per capita seem to have reached saturation for all product categories except construction. These trends are shown in Figure S 7, which were used to obtain the values in Table S 7.

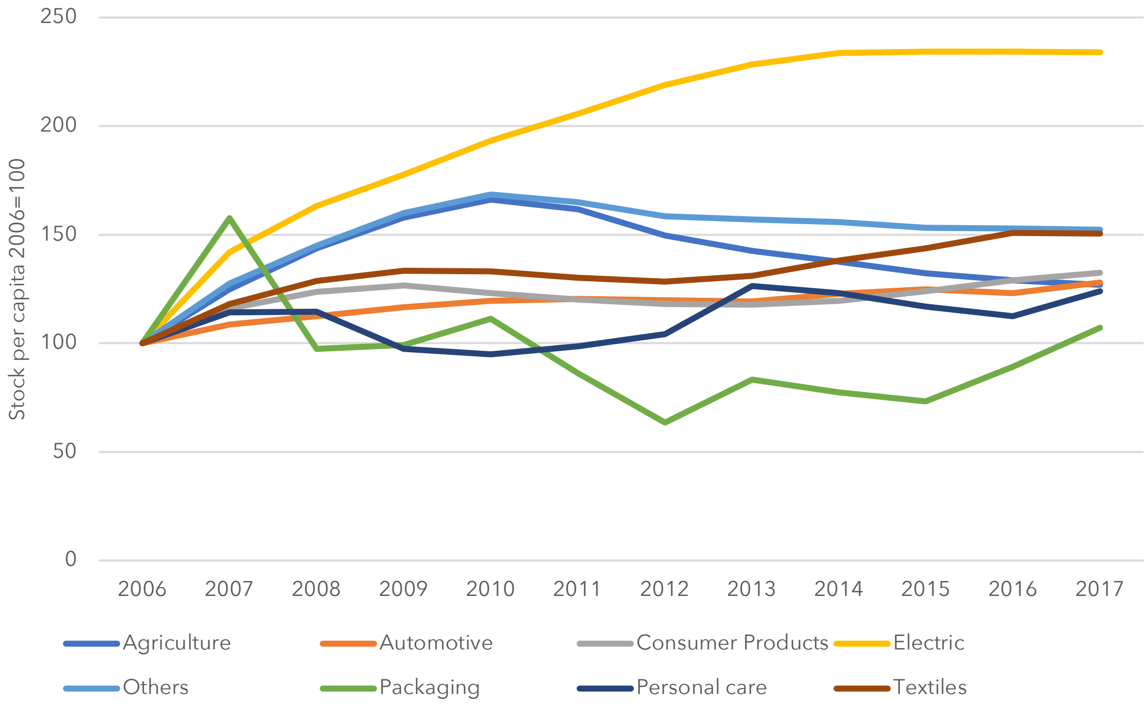

Figure S 7. Trends of stock per capita for various categories of plastics products.

For construction, the trends show that the stock has not yet reached saturation (see Figure S 8), most probably because our time series is not long enough to produce an accurate estimate of the current stock of construction plastics in service. For this reason, we used a different approach to estimate future stocks of construction plastics — we estimated the rate at which the construction stock has been accumulating in the UK (Figure S 9), and used a regressed trend to estimate the annual growth rate of the stock per capita of plastics in construction until it reaches saturation (equation (5), where r is the annual growth rate and t is the year). Beyond 2028, where the annual growth rate is estimated to be 0, the saturation of stock per capita is assumed.

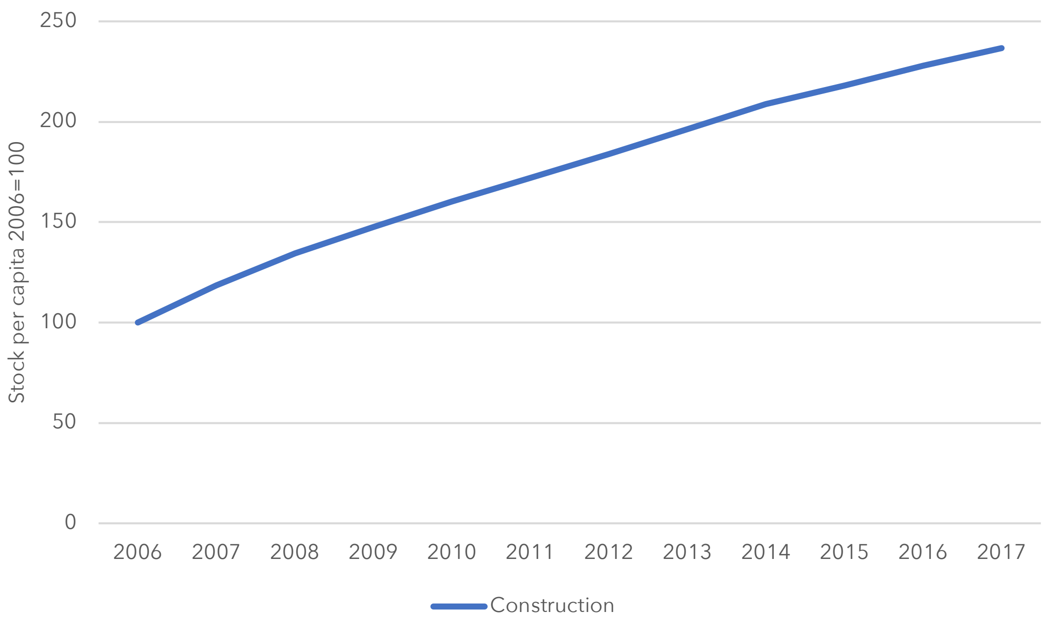

Figure S 8. Trends of stock per capita for plastics in construction.

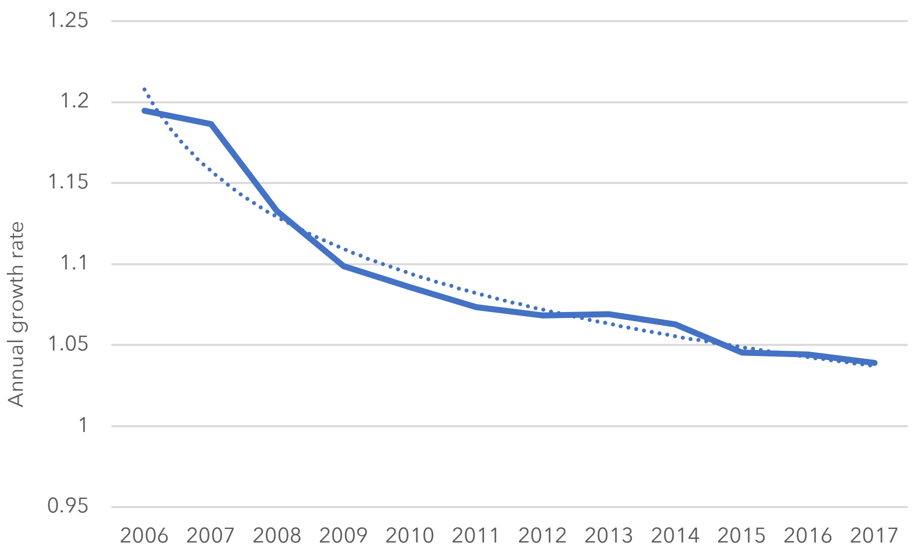

Figure S 9. Annual growth rate of the stock per capita of plastics in construction in the UK.

$$(1 + r) = 1.2078\,(t - 2006)^{-0.061} \quad (5)$$

6. Estimates of plastics demand and waste generation

Figure S 10 shows the estimated demand for plastics and waste generated broken down by application. This figure is equivalent to Figure 3 in the main text, which is broken down by polymer. Over the past few decades, the stock of plastics used in construction has been accumulating in service, noticeably by the

use PVC frames in windows. These uses are expected to have long service lives, and for this reason they are expected to lead to a substantial increase of construction plastic waste in the coming decades.

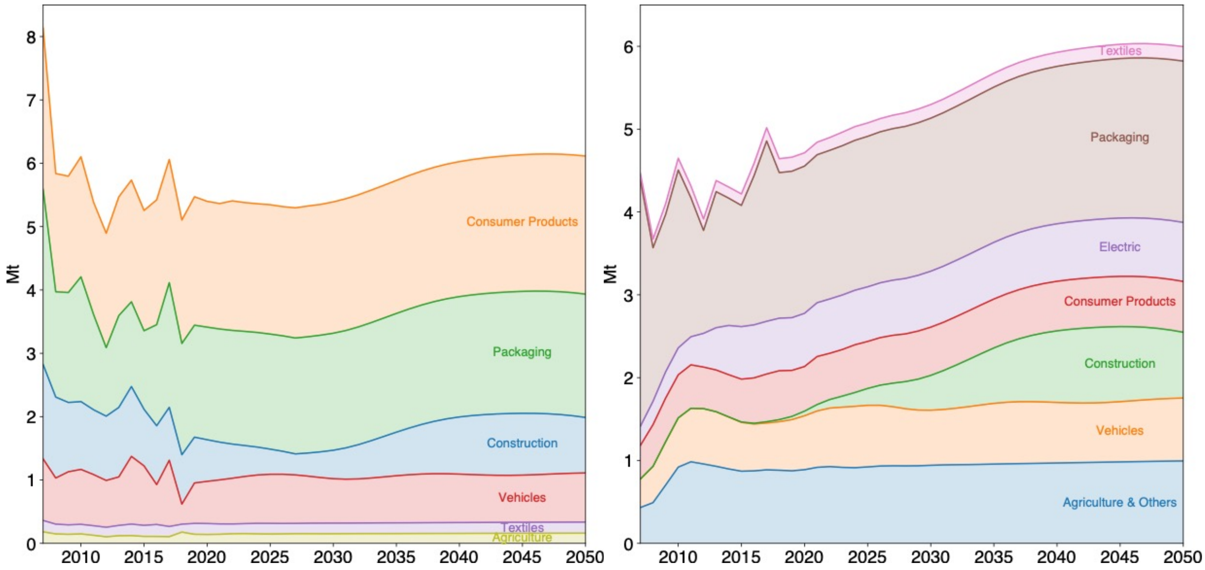

Figure S 10. Estimated demand for plastics in end-use products (left) and waste generated (right) in the UK by application.

7. Validation

In this section we compare the results of our analysis with existing literature providing estimates of plastics consumption and waste generation in the UK. For plastics consumption, we are only aware the recent study by WRAP (2018) on plastics packaging, and we show in Table S 8 the comparison of their estimate of plastics packaging placed-on-market with our estimation. For plastic waste generation there are various sources, and these are shown in Table S 9. In both cases, there is a good agreement between our and existing estimates.

Table S 8. Comparison of plastic packaging consumption in the UK in 2017.

	WRAP (2018)	This study
Packaging plastic (kt)	2 361	1 968

Table S 9. Comparison of plastic waste estimates for the UK in 2017.

	WRAP (2018)	Eunomia (2018)	This study
Packaging plastic (Mt)	2.4	3.5	2.2
Non-packaging plastic (Mt)	2.5	1.7	2.8
Total (Mt)	4.9	5.2	5.0

References

EUNOMIA. *Plastic Packaging – Shedding Light on the UK Data.* Eunomia, 2018. https://www.eunomia.co.uk/reports-tools/plastic-packaging-shedding-light-on-the-uk-data/.

EUROSTAT, Eurostat [online] Statistics on the production of manufactured goods (PRODCOM). 2018 [cited 02/05/2019]. Available from Internet: https://ec.europa.eu/eurostat/web/prodcom/data/database.

GEYER, R., JAMBECK, J.R., LAW, K.L. *Production, use, and fate of all plastics ever made.* Science Advances, 2017, **3** (7).

HMRC, HM Revenue & Customs [online] Trade statistics. 2019 [cited 11/02/2020]. Available from Internet: https://www.uktradeinfo.com/Pages/Home.aspx.

JAMBECK, J.R., GEYER, R., WILCOX, C., SIEGLER, T.R., PERRYMAN, M., ANDRADY, A., NARAYAN, R., LAW, K.L. *Plastic waste inputs from land into the ocean.* Science, 2015, **347** (6223): pp. 768.

ONS, Office for National Statistics [online] UK National Population Projections. 2017 [cited 10/11/2017]. Available from Internet: https://www.ons.gov.uk/peoplepopulationandcommunity/populationandmigration/populationprojections.

PLASTICSEUROPE, PlasticsEurope: Association of Plastics Manufacturers [online] European Plastics Industry Market Data. 2019 [cited 26/4/2019]. Available from Internet: https://www.plasticseurope.org/en/resources/market-data.

WRAP. *PlasticFlow 2025 — Plastic Packaging Flow Data Report.* WRAP, 2018. http://www.wrap.org.uk/sites/files/wrap/PlasticFlow%202025%20Plastic%20Packaging%20Flow%20Data%20Report_0.pdf.

WRAP. *Plastics market situation report 2019.* 2019. https://www.wrap.org.uk/plastics-market-situation-report-2019.
